# Water Oxidation by a Cytochrome P450: Mechanism and Function of the Reaction

**DOI:** 10.1371/journal.pone.0061897

**Published:** 2013-04-25

**Authors:** Brinda Prasad, Derrick J. Mah, Andrew R. Lewis, Erika Plettner

**Affiliations:** Department of Chemistry, Simon Fraser University, Burnaby, British Columbia, Canada; Instituto de Tecnologica Química e Biológica, UNL, Portugal

## Abstract

P450_cam_ (CYP101A1) is a bacterial monooxygenase that is known to catalyze the oxidation of camphor, the first committed step in camphor degradation, with simultaneous reduction of oxygen (O_2_). We report that P450_cam_ catalysis is controlled by oxygen levels: at high O_2_ concentration, P450_cam_ catalyzes the known oxidation reaction, whereas at low O_2_ concentration the enzyme catalyzes the reduction of camphor to borneol. We confirmed, using ^17^O and ^2^H NMR, that the hydrogen atom added to camphor comes from water, which is oxidized to hydrogen peroxide (H_2_O_2_). This is the first time a cytochrome P450 has been observed to catalyze oxidation of water to H_2_O_2_, a difficult reaction to catalyze due to its high barrier. The reduction of camphor and simultaneous oxidation of water are likely catalyzed by the iron-oxo intermediate of P450_cam_, and we present a plausible mechanism that accounts for the 1∶1 borneol:H_2_O_2_ stoichiometry we observed. This reaction has an adaptive value to bacteria that express this camphor catabolism pathway, which requires O_2_, for two reasons: 1) the borneol and H_2_O_2_ mixture generated is toxic to other bacteria and 2) borneol down-regulates the expression of P450_cam_ and its electron transfer partners. Since the reaction described here only occurs under low O_2_ conditions, the down-regulation only occurs when O_2_ is scarce.

## Introduction

**Figure 1:**
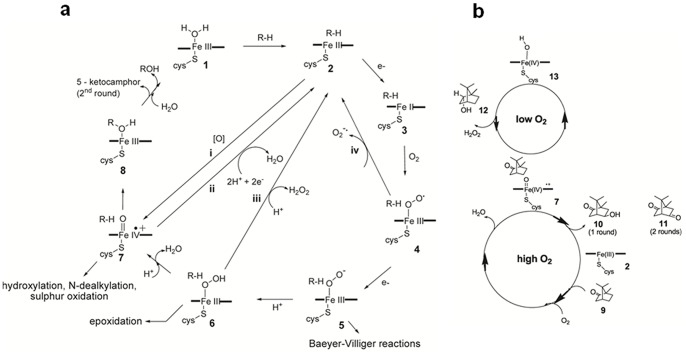
The catalytic cycle of P450_cam_ and the formation of the products, 10, 11 and 12. **a)** R-H represents the substrate, camphor. **i**, **ii**, **iii** and **iv** represent the peroxide shunt reaction, four-electron uncoupling, two-electron uncoupling, and the loss of superoxide. **b)** Under highly oxygenated conditions, P450_cam_ hydroxylates camphor **9** to 5-*exo*-hydroxy camphor **10** and further to 5-ketocamphor **11**, whereas under low oxygen conditions, P450_cam_ reduces camphor to borneol **12**.

Cytochrome P450 enzymes (P450s or CYPs) belong to a family of heme-thiolate enzymes that couple the reduction of oxygen to the oxidation of non-activated hydrocarbons [1]. The catalytic cycle of cytochrome P450_cam_ [2] (Fig. 1a) starts with binding of camphor to the resting enzyme **1** and expulsion of the axial water molecule to form **2**. Enzyme-substrate complex **2** accepts two electrons from the nicotinamide cofactor (NADH) *via* two redox partner proteins: an iron-sulfur protein, putidaredoxin (PdX), and a flavoprotein, putidaredoxin reductase (PdR) [3]. P450 utilizes the two electrons to reduce oxygen, O_2_, in a stepwise manner, *via* intermediates **3** and **4** [4], [5]. This leads to the formation of peroxo complex **5**, which is protonated to give hydroperoxo complex **6**. Protonation of the distal oxygen of **6** and elimination of water gives rise to a high valent iron-oxo complex **7** known as compound I (Cpd I) [6] (Fig. 1a). The oxygen from **7** is then inserted into a C-H bond of the substrate, giving an alcohol product complexed to the iron, **8**. The catalytic cycle is complete when water displaces the product.

Instead of proceeding through the complete reduction and splitting of O_2_, P450 enzymes can be shunted to Cpd I by using oxidants such as cumene hydroperoxide or *meta*-chloroperbenzoic acid (*m*-CPBA) (Fig. 1a, path “**i**”) [7], [8]. Furthermore, there are three alternate pathways that lead to uncoupling of NADH from camphor oxidation. First, Cpd I can be reduced by two electrons, and protonated twice giving the substrate complex **2** and water. This reductive pathway is known as four-electron uncoupling because it requires two NADH equivalents (Fig. 1a, path “**ii**”) [9], [10], [11]. Second, two-electron uncoupling is the dissociation of H_2_O_2_ (Fig. 1a, path “**iii**”) from the ferric hydroperoxo species **6**. Third, superoxide can dissociate from superoxo complex **4** (Fig. 1a, path “**iv**”) [1].

P450_cam_ (CYP101A1) enables a strain of *Pseudomonas putida* (a soil bacterium) to use (1R)-(+)-camphor **9** (Fig. 1) as a carbon source, and it oxidises camphor at the 5^th^ position to give 5-*exo*-hydroxycamphor **10** and 5-ketocamphor **11** (Fig. 1) [12]. Here we describe how P450_cam_ can oxidize water to H_2_O_2_ and simultaneously reduce camphor to borneol **12** (Fig. 1b) under low O_2_ conditions, and how borneol regulates the expression of the P450_cam_ system. Catalytic water oxidation is difficult to achieve, because the reaction is endothermic and has a large barrier. [13], [14], [15] To our knowledge, this is the first description of a cytochrome P450 oxidizing water.

We have observed that at low oxygen concentration, regardless of whether Cpd I forms *via* reduction of O_2_ or by shunting with oxidants, P450_cam_ not only produces the oxidation products **10** or **11**, but can also reduce camphor to borneol (Fig. 1b) [16]. We have interpreted this reaction to give *P. putida* an ecological advantage over other non-camphor metabolising bacteria because borneol is bactericidal to non-P450 containing bacteria, but not to *P. putida* [16]. In this paper, we present the mechanism of the camphor reduction reaction and the regulatory effect of borneol on the expression of P450_cam_.

## Materials and Methods

### I) Materials

All solvents were distilled prior to use. Nicotine adenine dinucleotide, reduced (NADH), dithiothreitol (DTT), lysozyme, DNase, RNase, vitamin B_1_, riboflavin, 5-aminolevulinic acid, hydrogen peroxide (used for assays), protease inhibitors leupeptin, aprotinin, and 4-(2-aminoethyl)-benzenesulfonyl fluoride, butylated hydroxytoluene (BHT), cytochrome P450 CYP3A4 (C-4982), superoxide dismutase (S5639), catalase (C-1345), glucose oxidase (G-2133) were purchased from Sigma. Ethylenediaminetetraacetic acid (EDTA) was purchased from Fisher Scientific. Ferrous sulphate (FeSO_4_) was purchased from Allied Chemical, Canada. Gas chromatography/mass spectrometry (GC-MS) was carried out on a Varian Saturn 2000 MS equipped with a 30-m SPB-5 column (Supelco, 0.25 mm ID; 0.25 µm film thickness) and the column was programmed as follows: 45°C (0.5 min), 7°C min^−1^ to 120°C (1 min), 50°C min^−1^ to 260°C (3 min). Electron impact (EI) spectra were obtained at an emission current of 30 µA, scanning from 50 to 365 amu, with ion storage (SIS mode) 49–375, trap temperature 170°C and transfer line 250°C. UV/Vis spectra were obtained on a Cary 300 Bio UV-visible double beam instrument. NADH utilization rates and hydrogen peroxide formation were measured on a thermostatted Hach DR/4000 U spectrophotometer. Activity assays were carried out at 22°C. Electrophoresis was performed on polyacrylamide gels (14%, 29∶1) with 0.5% SDS (SDS-PAGE). The samples were reduced by treating with 1 µL of DTT stock (31 mg/mL) before loading on gels. Gels were stained with Coomassie Brilliant Blue R (Sigma). Sonication was done using a Branson Ultrasonic sonicator. Centrifugations were carried out with a Beckmann Avanti J-26 XPI centrifuge, equipped with a JLA 8.1000 rotor.

The buffers used were: lysis (20 mM phosphate buffer (K^+^), pH 7.4 with 1 mM camphor; T-100 (50 mM Tris, 100 mM KCl, pH 7.4); T-400 (50 mM Tris, 400 mM KCl, pH 7.4). Buffers for nickel columns were: rinse buffer (20 mM Tris, pH 8.0); low imidazole buffer (5 mM imidazole, 20 mM Tris, 0.5 M NaCl, pH 8.0); strip buffer (0.1 M Ethylenediaminetetraacetic acid (EDTA)), 0.5 M NaCl, pH 8.0). For P450_cam_ purifications, all buffers contained 1 mM camphor. Substrate-free P450 was prepared by passing the substrate bound enzyme over a Sephadex G-10 column equilibrated with 100 mM 3-(N-morpholino) propane sulfonic acid (MOPS, pH 7.0).

### II) Methods

Deuterium (^2^H) NMR spectra were recorded on a Bruker AVANCE II 600 MHz spectrometer (operating at 92.124 MHz). A Bruker 5 mm TCI cryoprobe was used with samples maintained at a temperature of 298 K. ^2^H field-locking and field sweep were turned off. Samples were contained in 3 mm diameter MATCH nmr tubes filled to 40 mm (volume ca. 185 µL). Acquisition details: 10,240 transients summed, spectral width 15 ppm, transmitter offset 6.5 ppm, 11054 complex points acquired, 15 degree pulse with recycle delay of 1 s between transients, no decoupling of ^1^H during FID acquisition. Acquisition time was 14.2 h per spectrum.

The ^17^O NMR spectra were run on a Bruker AVANCE III 500 MHz NMR spectrometer (operating at 67.808 MHz) equipped with a Bruker 5 mm TBO probe and samples were maintained at a temperature of 298 K. Samples were contained in 5 mm diameter nmr tubes filled to 50 mm (volume ca. 600 µL). Acquisition details: 1,000 or 25,000 transients summed, spectral width 503 ppm, ^17^O transmitter offset 50 ppm, ^1^H transmitter offset 4.78 ppm, 32768 complex points acquired, 90 degree pulse with recycle delay of 1 s between transients, and inverse-gated WALTZ-16 composite pulse decoupling of ^1^H during FID acquisition. Acquisition time was 12 min per spectrum or 300 min (when catalase was present). The chemical shifts (δ) for all compounds are listed in parts per million using the NMR solvent as an internal reference (0 ppm for ^17^O or 4.78 ppm for ^2^H).

### III) Protein Expression and Purification

*E. coli* strain BL-21 (DE 3) (Novagen) containing the appropriate plasmid [17] were grown in Luria Broth-ampicillin (LB-amp) medium at 37°C with shaking (250 rpm) to A_600_ = 0.9–1.0 [17]. At this point, cells were harvested by centrifugation, resuspended in fresh LB-ampicillin medium, and after 2 h of growth, IPTG (240 mg L^−1^) and trace additives were added. The cultures, except for PdR, were grown for 12 h at 27°C (PdR was grown for 6 h). The cells were harvested by centrifugation (30 min, 7000×g) and stored at −85°C until lysis. Additives were: FeCl_2_ (0.1 µM), 5-aminolevulinic acid (1 mM), Vitamin B_1_ (10 µM) for P450; FeCl_2_ (0.1 µM) and Na_2_S.9H_2_O (0.1 µM) for redoxin; riboflavin (1 mM) for reductase.

The lysis steps of P450 and PdR remained the same as described for *P. putida* [10] except that 1 mM camphor was added to the buffer in which P450 culture was lysed. The dialysed lysate of P450, or PdR was individually subjected to a 20% ammonium sulphate cut to remove the cell debris. The 20% supernatant was then carried forward to 45% ammonium sulphate saturation to isolate the protein. The 20–45% pellet was resuspended in T-100 buffer (50 mM Tris, 100 mM KCl, pH 7.4), camphor (1 mM) was added in the case of P450 and purified by DE-52 (anion exchange column) using a linear gradient with buffer T-100 to T-400, 1 mM camphor and 1 mM β-mercapto ethanol (P450 only) at 1 mL min^−1^. The fractions with high absorbances at λ_392_ (in the case of P450), λ_454_ (in the case of PdR) were checked with SDS-PAGE. The collected fractions were pooled and concentrated using an Amicon ultrafiltration cell equipped with a YM-10 membrane and the concentrated protein was individually loaded onto a S-100 column, eluted with T-100 buffer, 1 mM sucrose, 1 mM camphor (P450 only) at 1 mL min^−1^. SDS-polyacrylamide gel electrophoresis showed a single band for P450 and PdR.

In the case of PdX, cells from 2 L of culture were lysed in lysis buffer (0.25 M NaCl, 20 mM Tris/HCl, pH 8.0). Lysozyme (10 mg mL^−1^), DNase (2 mg, Sigma), and RNase (10 mg, Sigma) were added and the solution was stirred for 30 min at 4°C. The lysate was sonicated with 50% duty cycle for 10 minutes, stirred for 10 min at 4°C, and homogenized with a pestle. The homogenized cells were then harvested by centrifugation (10500×g, 30 min) and dialysed with frequent changes of lysis buffer followed by further purification by ammonium sulphate precipitation. The dialysed lysate was subjected to a 20% ammonium sulphate cut to remove the cell debris. The 20% supernatant was then carried forward to 90% ammonium sulphate saturation overnight to isolate the protein. The 20–90% pellet was resuspended in 5 mL of rinse buffer (20 mM Tris HCl, pH 8.5), dialysed against this buffer for 3 h and harvested at 5000 rpm for 5 min. The dialysed supernatant was loaded on a ∼5 cm Ni^2+^-His bind column and eluted with strip buffer (10 mL×3), low imidazole buffer (10 mL×2), high imidazole buffer (10 mL×4). The fractions with A_280_/A_325_<5.0 were pooled, dialysed with 100 mM Tris, 100 mM KCl, pH 7.4 and the concentrated PdX protein was frozen to −85°C. The concentrations of ferric P450 (with camphor), PdR and PdX were determined by their extinction coefficients (ε_392_ = 68.5 mM^−1^ cm^−1^, ε_454_ = 10 mM^−1^ cm^−1^, ε_325_ = 15.6 mM^−1^cm^−1^ respectively).

The procedures for the enzymatic assays with the recombinant proteins, as well as the superposition and docking procedures, using Molecular Operating Environment (MOE, Montréal, Canada) are included in Material S1.

## Results and Discussion

### I) Reaction Conditions Leading to Formation of Borneol

We have observed that borneol forms as a major product of P450_cam_ when camphor is present and the O_2_ concentration is low (O_2_≤2 mg/L, ≤63 µM). *In vivo*, this occurs when cultures are poorly aerated [16] and, *in vitro*, this occurs when the buffer is sparged with argon in an open vial. In contrast, the known oxidation products **10** and **11** form at high O_2_ concentrations (∼9 mg/L = 284 µM). *In vivo*, this occurs when cultures are well aerated [16] and, *in vitro*, this occurs when pure O_2_ is bubbled into the buffer (Fig. 1b). To map the mechanism of the reduction, we have performed experiments with the recombinant proteins (P450_cam_, PdX, and PdR), isolated from expression in *E. coli* (Table 1). Assays were carried out in phosphate buffer (50 mM phosphate, 150 mM K^+^, pH 7.4), with NADH and camphor. Our extinction coefficient values were used for the calculation of the enzyme concentration (Table S1). Under high oxygenation (with pure O_2_ bubbled into the buffer), we observed 5-*exo*-hydroxy camphor as a major product (Table 1, entry 1). Similar experiments under mid-range oxygenated conditions (with air-treated buffer) yielded borneol as the only product (Table 1 entry 2). The formation of borneol under these conditions was 34-fold less compared to 5-*exo*-hydroxy camphor that formed under highly oxygenated conditions, and this could be because of the slower formation of iron-oxo species (Compound I).

**Table 1:** Assays with recombinant proteins: Formation of borneol, 5-ketocamphor and 5-*exo-* hydroxy camphor under various conditions.

Enzymatic assay	Products (nmol min^−1^nmol^−1^ P450)			NADH consumed (nmol min^−1^ nmol ^−1^ P450)	H_2_O_2_ formed (nmol min^−1^ nmol ^−1^ P450)	4e^−^ uncoupling (nmol min^−1^ nmol^−1^ P450)
	Borneol	5-keto camphor 11	5-*exo*-hydroxy camphor 10			
O_2_ 1	7.5±4	20±5	950±465	1331±270	ND	660±235
air^2^	28±9	ND	ND	335±13	297±103	80±10
rP450+ *m*-CPBA^3^	249±28	ND	ND	N/A	291±29	N/A
Ar+rP450+ *m*- CPBA^3,4^	404±19	16±4	ND	N/A	444±16	N/A
O_2_+ rP450+ *m*-CPBA^3,5^	173±39	354±12	ND	N/A	204±17	N/A

Values are the average of 4 replicates ± S.E. 50 mM potassium phosphate buffer (pH 7.4) was used for all the assays. Experimental details are included in Material S1.

ND = Not Detected; N/A = Not Applicable.

1 The reaction mixture contained recombinant P450_cam_, PdR and PdX and NADH. Oxygen (99%) was bubbled into the buffer for 60 seconds before the assay. The 4e^−^ uncoupling was calculated by taking the difference between the total NADH required and observed. ^2^ The reaction mixture contained recombinant P450_cam_, PdR, PdX, and NADH. Air (charcoal filtered) was bubbled into the buffer before the assay. ^3 ^The assay was performed using recombinant P450_cam_ and *m*-CPBA as a shunt agent. ^4 ^The buffer was sparged with argon (99%). ^5^ The buffer was treated with oxygen (99% pure, Sigma Aldrich) and assays were performed using camphor.

Under poor buffer oxygenation, in the absence of NADH, P450_cam_ shunted with *m*-CPBA (Fig. 1a, pathway “**i**”) reduced camphor to borneol (Table 1 entries 3 and 4). The observation that borneol formed in the absence of NADH indicates that NADH is not the source of electrons for the reduction reaction. Furthermore, shunted P450_cam_ under high buffer oxygenation gave more 5-ketocamphor than borneol (Table 1 entry 5), indicating that O_2_ levels are important in the regulation of the reaction catalyzed by the enzyme.

### II) Source of the 2-H in Borneol

**Figure 2:**
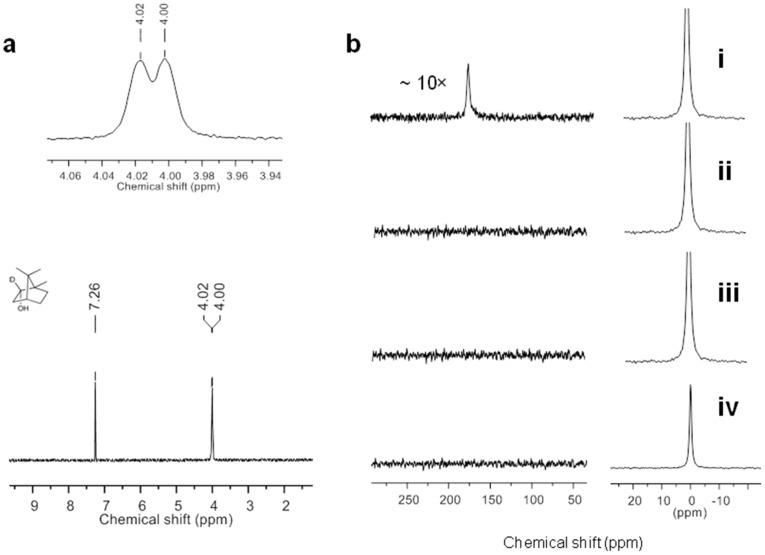
^2^H NMR of the 2-D-borneol and ^17^O NMR in the detection of H_2_^17^O_2_. **a)** ^2^H NMR of the 2-D-borneol obtained from the recombinant proteins incubated in 50 mM deuterated phosphate buffer (pD = 7.4) with camphor and *m*-CPBA. The extracted product was backwashed with H_2_O. The peak at 7.26 ppm corresponds to CHCl_3_ in CDCl_3_. **b)** ^17^O NMR spectrum of the incubation mixture in ^17^O phosphate buffer (pH 6.3) containing: **i)** camphor, recombinant P450_cam_ and *m*-CPBA, **ii)** camphor and recombinant P450_cam_ (*m*-CPBA absent), **iii)** camphor and *m*-CPBA (enzyme absent), and **iv)** *m*-CPBA and recombinant P450_cam_ (substrate absent). The peaks at 0 ppm and 178 ppm correspond to H_2_^17^O and H_2_^17^O_2_, respectively.

Because NADH is not the source of electrons for the reduction of camphor, the source of the hydrogen attached to C-2 of borneol was further investigated in assays using deuterated phosphate buffer (50 mM phosphate in D_2_O, 150 mM K^+^, pD 7.4). Using recombinant proteins (P450_cam_, PdR, and PdX), under mid-range oxygenated conditions (with air), we detected the enzymatic conversion of camphor to 2-D-borneol **12D** (Fig. 2a, Table S2) using ^2^H NMR. We also detected 5-ketocamphor, as well as the depletion of NADH (Table S2). Similar experiments using NADD (deuterated nicotinamide cofactor) in non-labeled phosphate buffer did not yield 2-D-borneol [18]. Enzymatic assays in deuterated buffer (with recombinant P450_cam_, shunted with *m*-CPBA, in the absence of NADH) also yielded borneol that was deuterated at C-2 (H_exo_) (Fig. 2a, Table S2). All these experiments lead to the conclusion that water is the source of H_exo_ attached to C-2 in borneol formed by P450_cam_.

### III) ^17^O NMR of H_2_O_2_

If camphor is reduced to borneol by electrons from water, then water should be oxidized to hydrogen peroxide. We observed H_2_O_2_ along with borneol, approximately in a 1∶1 stoichiometric ratio when P450_cam_ was shunted with *m*-CPBA (Table 1, entries 3–5) or with other oxidants (Table S5). We prepared H_2_^17^O [19] and incubated the reaction mixture containing 1 mM camphor, 1 mM *m*-CPBA and recombinant P450_cam_ (0.1 µM) in ^17^O phosphate buffer (50 mM, 150 mM K^+^, pH 7.4 made with H_2_^17^O) for 12 h to detect the formation of H_2_^17^O_2_. To this assay mixture, P450_cam_ (0.02 µM) and *m*-CPBA (0.2 mM) were added at 2 h intervals, to form detectable amounts of H_2_^17^O_2_. A new resonance was observed at 178 ppm in the ^17^O NMR spectrum, (Fig. 2b(i)) which matched the chemical shift of H_2_^17^O_2_ reported in the literature [20] and of our prepared standard [19]. The effect of pH on the chemical shift of hydrogen peroxide was also checked (Fig. S1). Controls (in the absence of *m*-CPBA, enzyme or substrate) were run simultaneously, and this resonance was not detected (Figs. 2b(ii), 2b(iii) and 2b(iv)), which led us to conclude that the new peak could not have come from the hydrolysis of *m*-CPBA. When catalase (an enzyme that disproportionates H_2_O_2_ to water and O_2_) was added to the reaction mixture, the resonance at 178 ppm disappeared (Fig. S2 b), confirming that the 178 ppm resonance is due to H_2_^17^O_2_.

### IV) Kinetic Isotope Effects (KIE)

**Figure 3:**
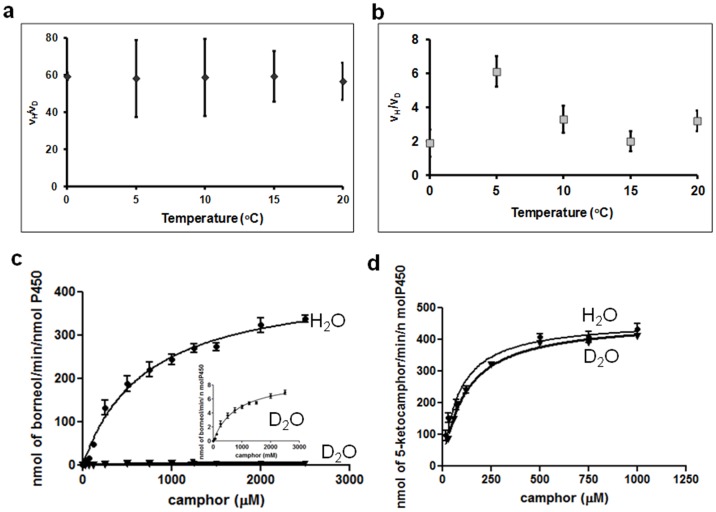
The Kinetic Isotope Effects for borneol and H_2_O_2_ and the Michaelis-Menten kinetics in their formation. **a)** Ratios v_H_/v_D_ at different temperatures for borneol and **b)** for H_2_O_2_ formation. **c)** Michaelis-Menten kinetics for borneol and **d)** 5-ketocamphor formation, under shunt conditions (with *m*-CPBA). To ensure a constant high O_2_ concentration for the 5-ketocamphor formation kinetics, reactions were run in vials fitted with septa and pressurized with pure O_2_.

The reaction catalyzed by P450_cam_, shunted with *m*-CPBA in D_2_O, gave 2-D-borneol at a much slower rate than the same reaction performed in normal water. The magnitude and temperature independence of the ^1^H/^2^H kinetic isotope effect (KIE) of ∼50 (Fig. 3a, Table S3) suggests that hydrogen transfer through tunnelling could occur at the rate-determining step in the reduction of camphor to borneol [21], [22], [23]. In contrast, the KIE (^1^H/^2^H) for hydrogen peroxide formation are much smaller, suggesting that this product does not form at the rate-limiting step (Fig. 3b, Table S4).

### V) Reduction Mechanism

Borneol formation under shunt conditions is saturable, with a *K*_M_ = 699±88 µM and *k*_cat_ = 426±20 min^−1 ^for camphor (Fig. 3c). Similarly, ketocamphor formation under oxygenated shunt conditions is saturable with a *K*_M_ = 83±10 µM and *k*_cat_ = 461±14 min^−1 ^for camphor (Fig. 3d). In D_2_O buffers, the formation of D-borneol was saturable with a *K*_M_ = 802±107 µM and *k*_cat_ = 9±0.4 min^−1 ^for camphor (Fig. 3). Ketocamphor formation under oxygenated shunt conditions is saturable with *K*_M_ = 118±6 µM and a similar *k*_cat_ = 465±6 min^−1 ^for camphor (Fig. 3). From control experiments we know that reducing P450_cam_ and camphor with dithionite does not yield any borneol (Fig. S3). Therefore, borneol formation requires oxidation of P450_cam_, either through shunting or through intermediates **2** to **7** of the catalytic cycle (Fig. 1a). Therefore, Cpd I must be involved in both borneol and ketocamphor formation (Fig. 1a).

**Figure 4:**
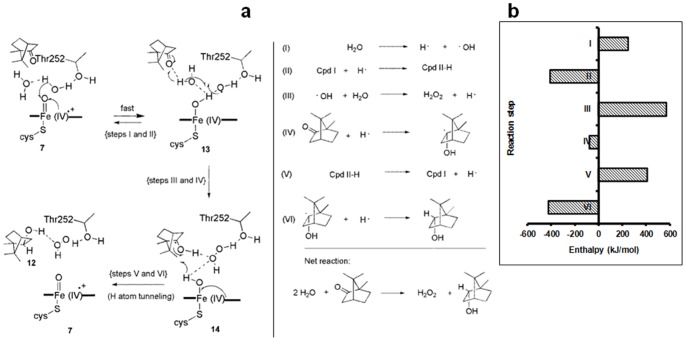
The proposed reduction mechanism and the Born-Haber estimates in the mechanism. **a)** Proposed reduction mechanism of P450_cam_ that accounts for the simultaneous formation of borneol **12** and H_2_O_2_, in a 1∶1 stoichiometry. **b)** Born-Haber cycle estimates of the reduction mechanism.

We propose that water reduces and protonates Cpd I as a first step in the borneol cycle, giving protonated Cpd II **13** and a hydroxyl radical (OH^•^) (Fig. 4). The formation of OH^•^ in water has been estimated from electrochemical data [24], and the formation of species **13** from Cpd I has been estimated at Δ*G*° = –410 kJ/mol [25]. Therefore, the first step of the proposed reduction mechanism (Steps I and II, Fig. 4) involves the abstraction of a hydrogen atom from water by Cpd I to form the Fe(IV)-OH complex **13** (Fig. 4), which is favourable (ΔH∼−160 kJ/mol) (Fig. 4). Three water molecules are known to be poised above the Fe-porphyrin and are held in place by hydrogen bonds to Thr 252, Asp 251 and Glu 366 [26], so it is plausible that Cpd I could attack water instead of camphor. Next, we propose that the hydroxyl radical combines with the water molecule to yield hydrogen peroxide and a hydrogen atom (Step III). By our estimate, this step is highly unfavourable (*ΔH*° ≅ 570 kJ/mol, Material S1, section 2.9). Simultaneous transfer of the hydrogen atom from step III to the carbonyl group of camphor forms a borneol radical (Step IV**,** Fig. 4). A non-strained ketone such as acetone reacting with a hydrogen atom has a potential of approximately −2 V (Δ*G*° is +173 kJ/mol) [27]. However, because camphor is quite a strained ketone, and that strain is relieved by the reduction, we have estimated this reaction to be slightly favourable (Δ*H*°∼−79±8 kJ/mol, Material S1, section 2.9). Finally, the transfer of a hydrogen atom from protonated Cpd II to the borneol radical forms borneol and Cpd I (Steps V and VI, Fig. 4) (Δ*H*° ≅ 13 kJ/mol), completing the “borneol cycle”. The net reaction is endothermic, with Δ*H*° ≅ 305±8 kJ/mol (Material S1, section 2.9, Fig. S4a).

The involvement of OH-radicals in H_2_O_2_ formation has been proposed previously for electrolytic catalysts that oxidize water to O_2_ (via an intermediate peroxide) [13] and also for a recently discovered water oxidation catalyst that produces H_2_O_2_ during electrolysis [14], [15]. Interestingly, the latter MnO_x_ catalyst stops the water oxidation process at H_2_O_2_, because the peroxide is solvated and stabilized by hydrogen bonding to ethylamine and/or water in the electrolyte. [14], [15] Analogously, here we propose that hydrogen bonding within the water cluster in the hydrophobic P450_cam_ active site is essential for stabilization of the various reactive intermediates and of the H_2_O_2_ formed. The turnover numbers with regard to H_2_O_2_ formation we have observed are ∼7, whereas the electrocatalytic systems give turnover numbers of 20–1500 for complete water oxidation to O_2_. [13] This difference arises because P450_cam_ only has access to thermal energy to perform this “uphill” reaction, whereas the electrocatalytic systems are run at overpotentials. [13], [14], [15].

The proposed mechanism accounts for our observation that borneol and hydrogen peroxide form in a 1∶1 stoichiometric ratio, provided 2-electron uncoupling is negligible (Table 1, Table S5). Given that Cpd I appears to be involved in the borneol cycle, our previous [16] and current data also suggest that Cpd I might be regulated by O_2_ levels: under high oxygenation, Cpd I sequentially hydroxylates camphor to **10**, or **10** to **11** (Fig. 1b). Under poor oxygenation, Cpd I enters the borneol cycle that couples the oxidation of water to H_2_O_2_ to the simultaneous reduction of camphor to borneol (Fig. 1b). The borneol cycle is independent of how Cpd I is generated: through the reduction of O_2_ or the shunt pathway (Fig. 1a). Borneol formation was seen with all the shunt agents tested (*m*-CPBA, cumene hydroperoxide, periodate and bleach; Table S5).

**Figure 5:**
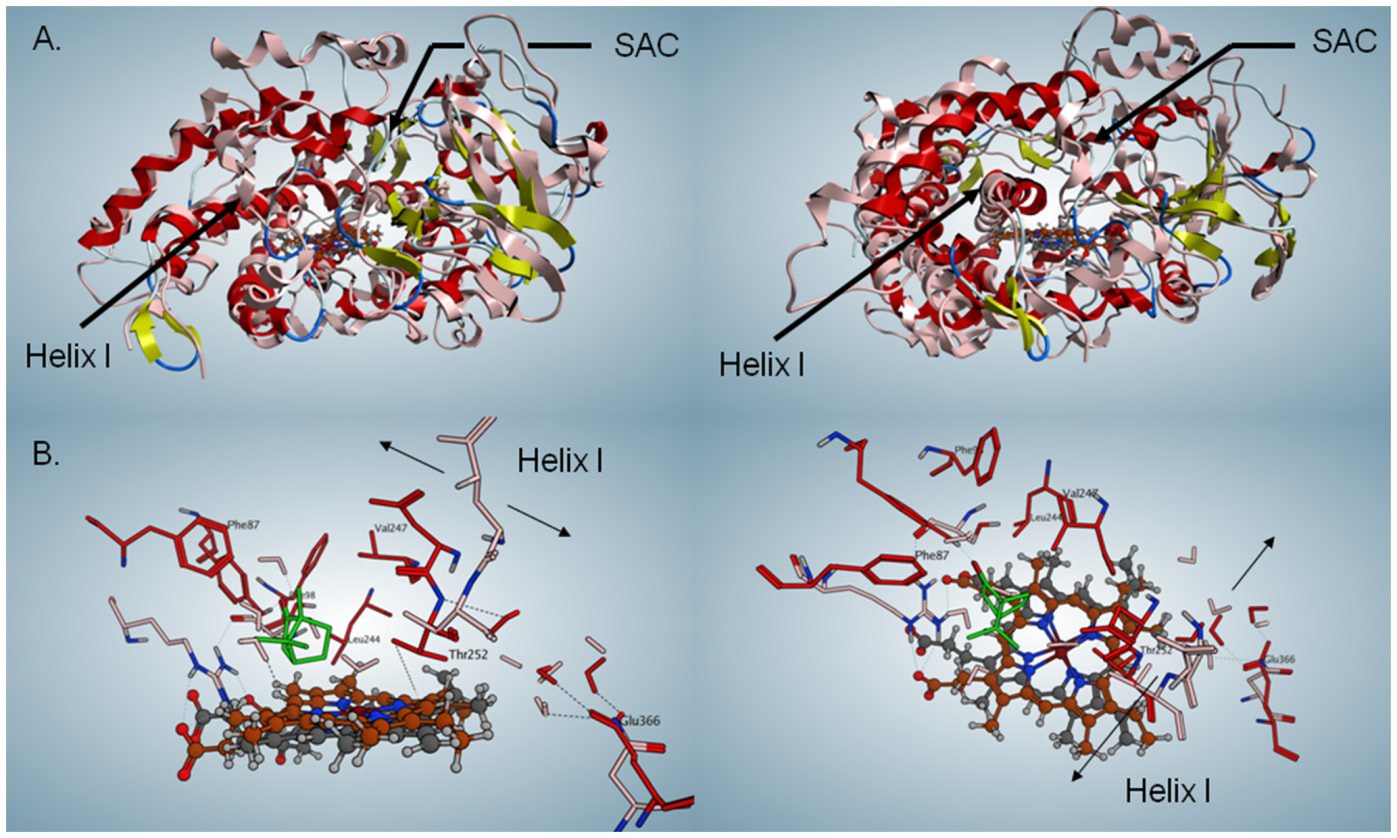
Superposition of P450_cam_ and CYP3A4. **a)** Top row: superimposed ribbon diagrams of P450_cam_ (1DZ4) and CYP3A4 (1TQN). P450_cam_ is shown with red helices and yellow sheets, whereas CYP3A4 is shown all in pink. The porphyrin for P450_cam_ is shown in gray and the one for CYP3A4, in brown. The two views are orthogonal to each other. The substrate access channel (SAC) is marked, as is Helix I, the central pillar of the fold. **b)** Lower row: superimposed active sites of P450_cam_ and CYP3A4. The porphyrin of P450_cam_ is shown in gray, the one for CYP3A4 in brown. The camphor ligand of P450_cam_ is shown in green. Residues from the two proteins are red (P450_cam_) and pink (CYP3A4). The two views are orthogonal to each other.

**Figure 6:**
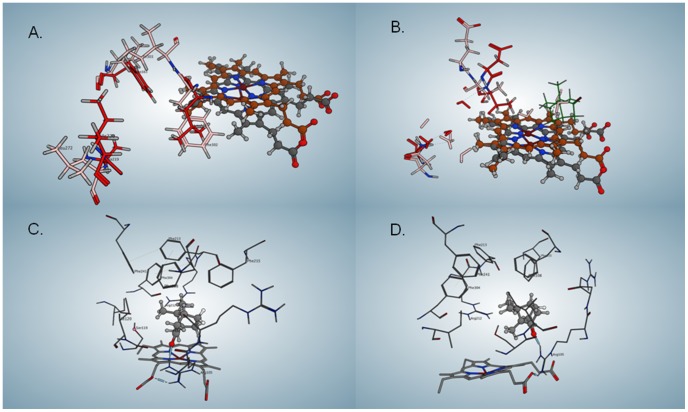
Sites in P450_cam_ and in CYP3A4 with camphor docked. a) Oxygen binding site in P450_cam_ (residues shown in red), with superimposed residues in CYP3A4 shown in pink. The porphyrin of P450_cam_ is gray, and the one for CYP3A4 is brown. b) Water channel in P450_cam_ (residues shown in red), with superimposed residues in CYP 3A4 shown in pink. The view in a) and b) are from a similar angle, to emphasize the closeness of the O_2_ binding site and the water channel in P450_cam_. c) and d) Camphor docked into the active site of CYP3A4 (orthogonal views). The H-bond from Arg 105 to the camphor ketone can be seen in the lower right portion of d).

In assays with CYP3A4 (a human cytochrome P450) under shunt conditions, 5-exo-hydroxy camphor formed as a major product. There were no detectable amounts of borneol, suggesting that the reduction cycle is specific to P450_cam_ (Material S1, section 2.5). A BLAST search against the P450_cam_ sequence revealed many other bacterial cytochromes P450 that show sequence identities for the three residues that hold a set of water molecules above the porphyrin (Asp 251, Thr 252 and Glu 366 in P450_cam_, Fig. S5), as well as for the hydrophobic residues that are involved in O_2_ binding (see below and Material S1, section 2.10). Superposition of P450_cam_ (1DZ4, [28] on CYP3A4 (1TZN, [29] reveals that the active site of CYP3A4 is much larger and more polar than that of P450_cam_. In P450_cam_, camphor is surrounded by closely packed hydrophobic residues, which could form a cage around the reactive intermediates. (Fig. 5) The only water in the active site of the camphor-bound structure is in the water channel between Glu 366 and Thr 252, whereas the CYP3A4 active site can hold numerous water molecules in the absence of a ligand (Fig. 6). Docking of camphor into the active site of CYP 3A4 reveals the camphor bound near the porphyrin, capped by five phenylalanine residues and surrounded by Arg 212, Ser 119, Ile 120, Ile 301 and H-bonded to Arg 105 (Fig. 6). This more open arrangement may not provide the necessary stabilization for water oxidation to occur. Furthermore, the different positioning of the camphor within the active site may also preclude its utilization as an electron acceptor during the water oxidation and, therefore, the reaction was not observed in CYP3A4.

Amunom *et. al.* have stated that mammalian P450s can reduce 4-hydroxynonenal to 1,4-dihydroxynonenal under low oxygen conditions, [30] similar to our results presented in this paper. However, differences in the reaction mechanisms can be associated with the different reacting species of the P450. They proposed that the reduction they observed occurs via the ferrous (Fe (II)) species of P450, where the electron source is from NADPH through the NADPH-P450 reductase. We have shown that, in our case, the ferrous species is not involved (Fig. 4) We therefore propose that the reduction of camphor to borneol involves the iron-oxo species where the source of electrons is from water, and not from NADH. Kaspera *et. al.* have stated that P450BM-3 from *Bacillus megaterium* can reduce *p*-methoxy-benzaldehyde to methoxy-benzalcohol [31]. Electrons for this reaction are provided by a direct hydride transfer from NADPH to the aldehyde, or by NADPH reduction of the flavin mononucleotide in the reductase, which then reduces the substrate. In comparison, we found that the source of electrons in our case is clearly from water, and not from a direct hydride transfer.

### VI) Control Experiments with Reactive O_2_ Species/quenchers

*In vitro* assays with P450 under shunt conditions were performed with a free radical quencher (BHT), a free metal chelator (EDTA), catalase and superoxide dismutase, to determine whether free reactive oxygen species are involved in borneol formation (Table 2). Under shunt conditions using *m*-CPBA, in Ar-sparged buffer, the enzyme formed more borneol than 5-ketocamphor (Table 1, entry 4; Table 2, entry 1). In the presence of catalase (Table 2, entry 2), the borneol formed was ∼50% lower due to the decomposition of H_2_O_2_ to O_2_. We confirmed that O_2_, but not H_2_O_2_, had an effect in lowering borneol formation by performing experiments with an O_2_ scavenging system (glucose/glucose oxidase) (Table 2, entry 3). With catalase alone, the O_2_ formed by the catalase-mediated decomposition of H_2_O_2_ regulated the enzyme such that it produced some ketocamphor. In contrast, in the presence of catalase and glucose oxidase/glucose, the O_2_ was destroyed and no ketocamphor formed. To check if superoxide plays a role in borneol formation, we performed experiments with superoxide dismutase and detected no significant effect in borneol formation (Table 2, entry 4). To check if the radicals proposed in the mechanism of the reduction (Fig. 4) can diffuse out of the P450’s active site, we experimented with BHT, and noticed no significant effect (Table 2, entry 5). This suggests that any radical species involved in the borneol cycle do not exist long enough to diffuse out of the active site of P450_cam_. To test if a metal impurity plays a role in our assays under shunt conditions, experiments were performed with EDTA, and we detected no effect on borneol formation (Table 2, entry 6). To check if free iron (outside of the active site) plays a role in reduction reaction, experiments were performed with ferrous sulphate and *m*-CPBA, in the absence of P450_cam_, and we did not detect borneol or 5-ketocamphor (Table 2, entry 7). These experiments suggest that the reduction of camphor to borneol is catalysed by P450_cam_ alone, does not involve any adventitious metal species outside of the P450 active site and does not involve the diffusion of reactive oxygen species, other than the product H_2_O_2_, out of the active site.

**Table 2:** Tests for involvement of free reactive oxygen species: formation of borneol, 5-ketocamphor and H_2_O_2_.*

Enzymatic assay	Products(nmol min^−1^nmol^−1^ P450)		Ratio of Borneol: 5-ketocamphor	H_2_O_2_ formed (nmol min^−1^ nmol ^−1^ P450)
	Borneol	5-keto camphor		
Ar+rP450+ *m*- CPBA^1^	443±41	21±2	23±3	494±16
Ar+rP450+ *m*- CPBA+catalase^2^	246±20	18±1	13±0.6	ND
Ar+rP450+ *m*- CPBA+glucose/glucose oxidase^3^	469±16	ND	N/A	ND
Ar+rP450+ *m*- CPBA+superoxide dismutase^4^	398±10	ND	N/A	428±34
Ar+rP450+ *m*- CPBA+BHT^5^	454±28	25±6	20±4	478±22
Ar+rP450+ *m*- CPBA+EDTA^6^	438±34	19±6	29±8	412±33
Ar+FeSO_4_+ *m*- CPBA^7^	ND	ND	N/A	ND

* The experiments in Table 2 (except for entry 4) were performed on February 20, 2013 when the GC-MS was more sensitive to borneol detection than previous assays, due to installation of a new electron multiplier.

Values are the average of 4 replicates ± S.E. 50 mM potassium phosphate buffer (pH 7.4) was used for all the assays and was sparged with argon (99%). Camphor was the substrate in all assays. Experimental details are included in Material S1.

ND = Not Detected; N/A = Not Applicable.

1 The assay was performed using recombinant P450_cam_ and *m*-CPBA as a shunt agent. ^2^ The assay was performed using recombinant P450_cam_, *m*-CPBA and catalase. ^3^ The assay was performed using recombinant P450_cam_, *m*-CPBA and glucose/glucose oxidase. ^4^ The assay was performed using recombinant P450_cam_, *m*-CPBA and superoxide dismutase. ^5^ The assay was performed using recombinant P450_cam_, *m*-CPBA and butylated hydroxytoluene. ^6^ The assay was performed using recombinant P450_cam_, *m*-CPBA and EDTA. ^5^ The assay was performed using recombinant P450_cam_, *m*-CPBA and butylated hydroxytoluene. ^6^ The assay was performed using recombinant P450_cam_, *m*-CPBA and EDTA. ^7^ The assay was performed using *m*-CPBA and ferrous sulphate.

### VII) Role of Oxygen in the Borneol Cycle

The reaction path taken by P450_cam_ (camphor oxidation *vs*. borneol cycle, Fig. 1b) is controlled by oxygen concentration. Oxygen could exert its effect in two ways: 1) by affecting the interaction of P450 with its redox partners, or 2) by directly interacting with P450. Our results demonstrate that the former cannot be the case, because the effect was seen in the absence of PdX and PdR (Table 1). Therefore, P450_cam_ must bind O_2_ not only for catalysis, but also for allosteric regulation.

Recently, cytochrome P450 2E1 has been shown to form endoperoxide rearrangement products when reacted with 1,1,2,2-tetramethylcyclopropane [32]. This suggests that there must be O_2_ bound in that enzyme near the active site, which reacts with the rearranged radical formed by H-atom abstraction from 1,1,2,2-tetramethylcyclopropane. Cytochromes P450 are known to be allosterically regulated by their substrates or co-substrates [33]. Studies with other O_2_-utilizing enzymes, such as diiron monooxygenases [34], laccase [35] and amine oxidase [36] have revealed that O_2_ can be bound in hydrophobic tunnels that are separated from the access channel for the other substrates of these enzymes. In P450_cam_, a hydrophobic O_2_ entry channel and two O_2_ binding cavities have been identified in Xe-treated crystals [37]. Two Xe atoms are bound near the porphyrin edge in a hydrophobic pocket lined by F163, A167, heme allyl, I220, A219, C242 and L245. The other two Xe atoms appear bound in a crevasse lined by L371, T370, L257, M261, water and S260 (first Xe) and I275, K372, T376, L375, L371, P278 and I281 (second Xe). This O_2_ binding site in P450_cam_ is located near the edge of the porphyrin, near the water channel (Fig. 6 and 1 A. and B).

We have found a hydrophobic tunnel in P450_cam_ that includes the Xe binding sites, using MOLEonline 2.0 [38] on the structure believed to represent the P450_cam_ oxo complex (1DZ9). The binding sites are good candidates for O_2_ binding because they are hydrophobic and distinct from the substrate access route [11], [37]. Also, the sites are good candidates for allosteric regulation of P450 because they are near the plane of the porphyrin. It is plausible that an O_2_ molecule bound near the heme could affect the reactivity of Cpd I.

The O_2_ binding site in P450_cam_ is closer to the porphyrin than the equivalent site in CYP3A4, and the O_2_ binding site is lined by different residues (Fig. 6). Furthermore, the O_2_ binding site in P450_cam_ is close to the water channel, the only source of water in the camphor-filled active site of P450_cam_. It is reasonable to hypothesize that the O_2_ site, the porphyrin, the water channel and the tightly held camphor, all of which are near each other, could affect each other by allosteric effects in P450_cam_.

It is interesting to note that the *K*_M_ for ketocamphor formation under high O_2_ concentration is 9-fold lower (see above) than that for borneol formation under low O_2_ concentration. This suggests that camphor binding and possibly positioning might be affected by O_2_ concentrations. Surprisingly, the *k*_cat_ is the same for both reactions, even though there appears to be a larger barrier in the borneol cycle than in the normal oxidation reaction. This larger-than-expected *k*_cat_ suggests that, consistent with the observed KIE, H-atom tunneling is occurring in the borneol cycle. Under high O_2_ concentrations using D_2_O as the solvent, 5-ketocamphor (Table S2) was detected as the only product suggesting that deuterium atoms from the solvent do not participate in that reaction. Steady-state kinetic assays for ketocamphor formation in D_2_O buffers resulted in similar *k*_cat_ as in H_2_O buffers. In contrast, a 60-fold decrease in *k*_cat_ (with a similar *K*_M_) was detected for borneol formation (Fig. 3). This illustrates that the solvent molecules participate only in the borneol formation, but not in ketocamphor formation.

There are two ways the cycle could end. Cpd I might oxidize a nearby enzymatic residue or, alternatively, the borneol radical might abstract a H-atom from water, giving borneol and OH^•^, and the hydroxyl radical could rebind with the OH^•^ bound in Cpd II-H, to give a second H_2_O_2_ and the ferric enzyme (Fig. S4 b).

### VIII) Adaptive Advantage of Borneol and H_2_O_2_ to *P. putida*

Previously we have determined the effect of borneol and camphor on the growth of *P. putida* and *E. coli* [16]. To determine the effects of hydrogen peroxide, we have tested the toxicity of H_2_O_2_ and a 1∶1 stoichiometric mixture of borneol and H_2_O_2_ on both *P. putida* and *E. coli*, a bacterium that lacks cytochrome P450 [39] (Figs. S6 and S7). The borneol/H_2_O_2_ mixture was lethal to *E. coli* and slightly toxic to *P. putida* (Fig. S7). The latter observation prompted us to explore whether borneol affects the expression of the P450_cam_ system.

**Figure 7:**
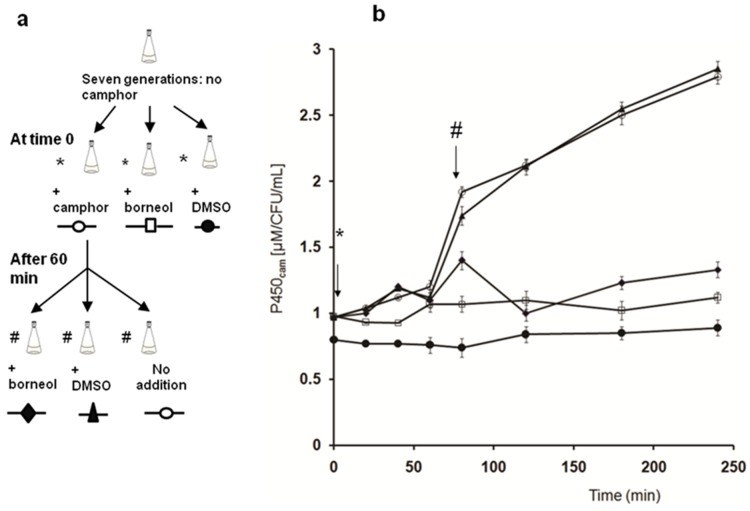
The effect of camphor, borneol and DMSO on the P450 expression. **a)** Outline of the experiment used to determine the effect of camphor and borneol on P450_cam_, PdX and PdR expression. **b)** The effect of camphor, borneol and DMSO on the P450 expression by *Pseudomonas putida* (ATCC 17453). The concentration of P450_cam_ was obtained from the Soret peak absorbances and was normalized against the number of colony forming units/mL. Points represent the average ± S. E. of three replicates.

The camphor metabolism pathway, of which P450_cam_ catalyzes the first step, is encoded on the Cam plasmid under the control of the Cam repressor. This repressor dissociates from the upstream control region of the Cam operon upon binding of camphor, ensuring that the entire operon is expressed when camphor is present [40]. To study this induction, we cultured *P. putida* in the absence of camphor for seven generations, then divided the culture and treated the sub-cultures as shown in Fig. 7a (with camphor, borneol or vehicle, dimethyl sulfoxide (DMSO)). We detected a steep increase in the characteristic absorption bands of P450_cam_, PdR, and PdX only in the culture induced with camphor, about 80 min after initial induction. Absorptions plummeted approximately 60 min after the addition of borneol to camphor-induced culture(s) (Fig. 7b, Figs. S8 and S9). This decrease in P450, PdR, and PdX expression must be due to the borneol addition, because the camphor-induced cultures that did not receive borneol expressed significantly higher levels of P450, PdR and PdX/CFU/mL than the borneol-treated cultures.

The borneol down-regulation of P450_cam_, PdX, and PdR might be advantageous to *P. putida* during periods of low soil aeration. Because the camphor degradation pathway requires four O_2_/camphor (to reach 5-hydroxy-3,4,4-trimethyl-2-heptenedioic acid-δ-lactone), and the P450_cam_-catalyzed oxidation is the first committed step [41], it is advantageous to regulate camphor metabolism at the first step. When aeration increases, low levels of P450_cam_ convert borneol back to camphor [16], and this frees the Cam operon from borneol down-regulation.

### Conclusions

We describe the borneol cycle of P450_cam_, a cycle that occurs at low O_2_ concentration. The cycle connects to the known catalytic cycle *via* Cpd I which is regulated by O_2_ levels: at low O_2_ concentration, Cpd I oxidises water, whereas at high O_2_ concentration, Cpd I oxidises camphor. Under low O_2_ concentrations, the slower formation of compound I and high energy barrier with tunneling could account for lower formation of borneol.

The Cpd I-catalyzed reaction of P450_cam_ proposed here (Fig. 4) is independent of the redox partner proteins (PdX and PdR) and of how Cpd I forms (O_2_ reduction or shunt). The reaction occurs both *in vitro* (this paper) and *in vivo* [16]. We show here that P450_cam_ couples the oxidation of water to H_2_O_2_ and the reduction of camphor to borneol. We have presented evidence that: i) water is the source of the 2-H in the borneol; ii) water is oxidized to form H_2_O_2_ when camphor is reduced; and iii) the transfer of an H atom from water to C-2 of camphor occurs at a rate-limiting step in the borneol cycle. We propose that the reactivity of Cpd I is regulated by O_2_ concentration, and we have located a potential access channel where O_2_ might bind to P450_cam_ to exert its allosteric control.

The borneol and H_2_O_2_ formed serve several ecological functions. First, borneol and H_2_O_2_ are not very toxic to *P. putida*, whereas the combination is lethal to bacteria such as *E. coli* which do not contain any P450 [16], and this may give *P. putida* an advantage in bacterial communities. Secondly camphor induces the expression of P450, PdX, and PdR, whereas borneol decreases the expression of these gene products in *P. putida*. These features of the P450_cam_ may protect the bacteria from excessive exposure to borneol and reactive oxygen species during prolonged periods of low oxygen concentration.

## Supporting Information

Figure S1 **^17^O NMR spectra of H_2_^17^O_2_ (obtained by electrolysis of H_2_^17^O) buffered at a) pH 10, b) pH 3, and c) pH 9.**
(TIF)

Figure S2 **^17^O NMR spectra of the incubation mixture under shunt conditions using 1 mM** ***m*** **-CPBA in ^17^O phosphate buffer (final pH 6.3) containing 1 mM substrate camphor and recombinant P450_cam_.**: **a**) before and **b**) after addition of catalase (1 unit). The peak at 178 ppm corresponds to H_2_^17^O_2_ and that at 0 ppm is due to H_2_^17^O.
(TIF)

Figure S3 **GC-MS traces of camphor incubated under shunt conditions using** ***m*** **-CPBA with: a) reduced P450_cam_, and b) with unreduced P450_cam_.** 1-indanone was used as an internal standard.
(TIF)

Figure S4 **a)** Summary of the borneol cycle steps and of the net reaction. **b)** Possible routes by which the borneol cycle could end.
(TIF)

Figure S5 **Alignment of microbial cytochromes P450 against P450_cam_ (upper portion) and of vertebrate class II P450s, also against P450_cam_ (lower portion).** Microbial sequences used: gamma prot 1 = marine gamma proteobacterium HTCC2207 (ZP_01225512), Novo ar CYP = *Novosphingbium aromaticivorans* CYP 101D2 (PDB 3NV6), Sphingo echi = *Sphingomonas echinoides* ATCC14820 (ZP_10341012), Novo CYP 101D1 = a camphor hydroxylase from *Novosphingobium aromaticivorans* DSM 12444 (PDB 3LXI), Sphing chlor = *Sphingomonas chlorophenolicum* camphor hydroxylase (ZP_10341012), Azospir B510 = *Azospirillium* sp. B510 (YP_003451823), Azospir = (BAI74843), P450 Burk H160 = *Burkholderia* sp. H160 (ZP_03264429), P450 Burk MCO-3 *Burkholderia cenocepacia* MC0-3 = (YP_001774494), Sping Witt R = *Sphingomonas wittichii* RW1 (YP_001262244), Citromicrobi = *Citromicrobium bathyomarinum* JL354 (ZP_06860768), Novo CYP 101 = *Novosphingobium aromaticivorans* DSM12444 CYP 101C1 (PDB 3OFT_C), Sping E 14820 = *Sphingomonas echinoides* ATCC 14820 (ZP_10339023), gamma prot 2 = marine gamma proteobacterium NOR51-B (ZP_04956740), Sphingomonas = *Sphingomonas* sp. KC8 (ZP_09138048), Sphing chl L = *Sphingobium chlorophenolicum* L-1 (YP_004553185), P450 nor = Cytochrome P450nor from *Fusarium oxysporum* (BAA03390). Vertebrate P450s: Cyp lan deme = lanosterol 14-α demethylase isoform 1 precursor *Homo sapiens* (NP_000777), CYP 2C9 = human liver limonene hydroxylase (P11712), CYP 4A11 *Homo sapiens* (NP_000769), CYP 4F12 = fatty acyl Ω-hydroxylase *Homo sapiens* (NP_076433), CYP 4F2 = leukotriene-B(4) omega-hydroxylase 1 precursor *Homo sapiens* (NP_001073), CYP 3A5 form 1 = CYP 3A5 isoform 1 *Homo sapiens* (NP_000768), CYP 3A4 = CYP 3A4 isoform 1 *Homo sapiens* (NP_059488), CYP26B1 = retinoic acid hydroxylase *Homo sapiens* (NP_063938).
(TIF)

Figure S6 **IC_50_ determination of a) H_2_O_2_ and b) of a 1∶1 (molar) mixture of borneol and H_2_O_2_ against** ***E. coli*** **, a species of bacterium that lacks cytochrome P450.**
(TIF)

Figure S7 **Effect of 16 h incubation of stationary** ***E. coli*** **(a)** ***and P. putida*** **(b) cultures with borneol: H_2_O_2_ (1∶1), borneol, or H_2_O_2_ (1 mM).**
(TIF)

Figure S8 **Expression profile of PdX in** ***P. putida*** **, in the presence and absence of camphor or borneol (see experimental map and symbols in** **Fig. 7a** **).** Points represent the average ± S. E. of three replicates.
(TIF)

Figure S9 **Expression profile of PdR in** ***P. putida*** **, in the presence and absence of camphor or borneol (see experimental map and symbols in** **Fig. 7a** **).** Points represent the average ± S. E. of three replicates.
(TIF)

Table S1 **Calculated and literature values of P450_cam_ extinction coefficients at selected wavelengths.**
(DOC)

Table S2 **Formation of 2-D-borneol and 5-ketocamphor in D_2_O buffer, with the full P450_cam_ system and with the shunted P450_cam_.**
(DOC)

Table S3 **Assays with recombinant proteins at selected temperatures. Formation of borneol, D-borneol, under shunt conditions with the addition of** ***m-*** **CPBA.**
(DOC)

Table S4 **Assays with recombinant P450_cam_, shunted with** ***m*** **-CPBA in H_2_O and D_2_O at selected temperatures. Formation of H_2_O_2_ or D_2_O_2_.**
(DOC)

Table S5 **Formation of borneol and hydrogen peroxide from the P450 catalytic cycle using several shunt agents.**
(DOC)

Material S1 **Supporting information for this paper.** This file contains detailed descriptions of various assays, calculations, sequence alignments and P450_cam_ system induction experiments. In addition, there are 9 supplemental Figures and 5 supplemental Tables.
(DOC)
