# Supplementary figures and images for: Water Oxidation by a Cytochrome P450: Mechanism and Function of the Reaction
Source: PLoS One. 2013 Apr 25;8(4):e61897. doi: 10.1371/journal.pone.0061897 (PMC3636257; doi:10.1371/journal.pone.0061897)

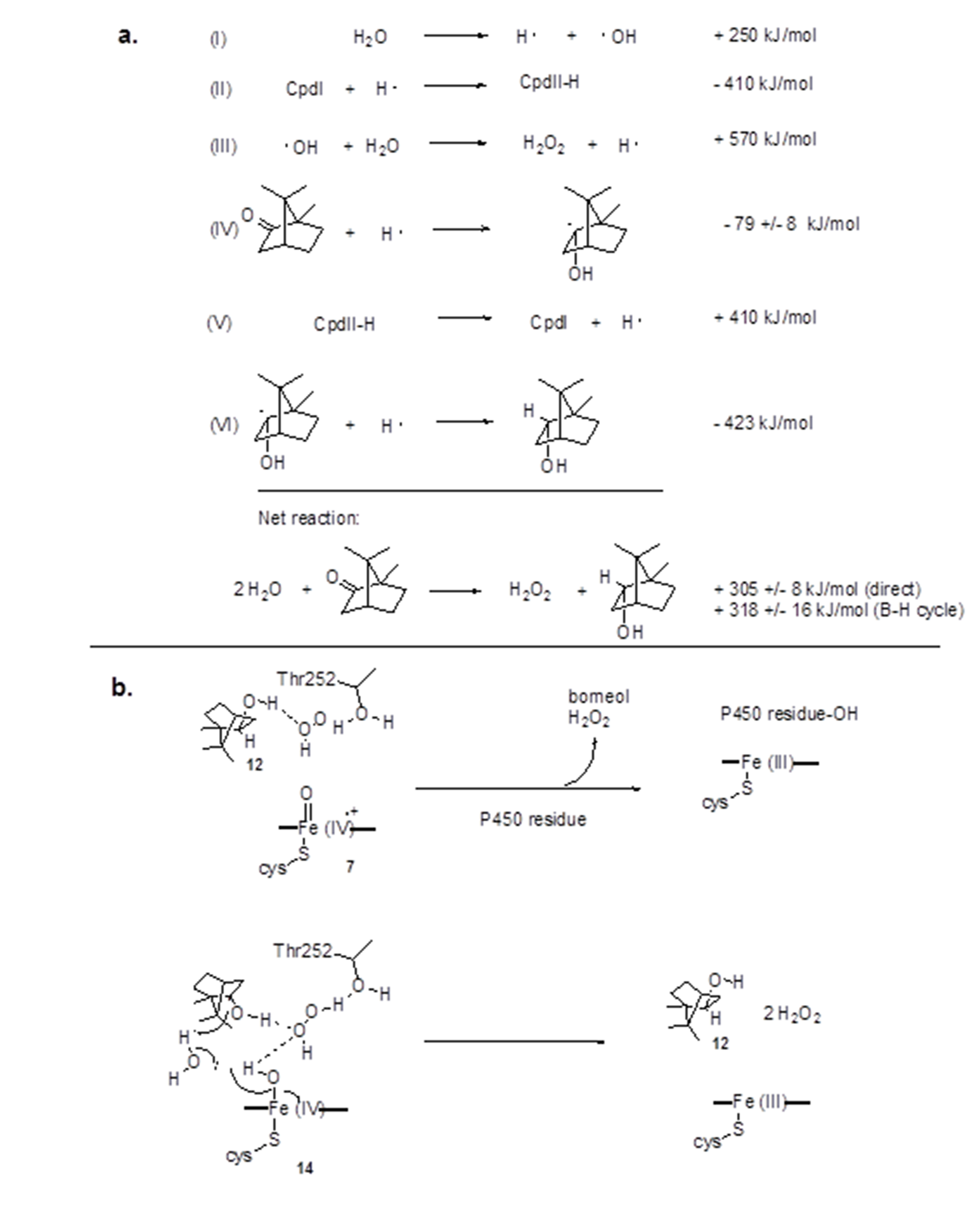

Supplement: Figure S4 — a) Summary of the borneol cycle steps and of the net reaction. b) Possible routes by which the borneol cycle could end. (TIF) [file pone.0061897.s004.tif]

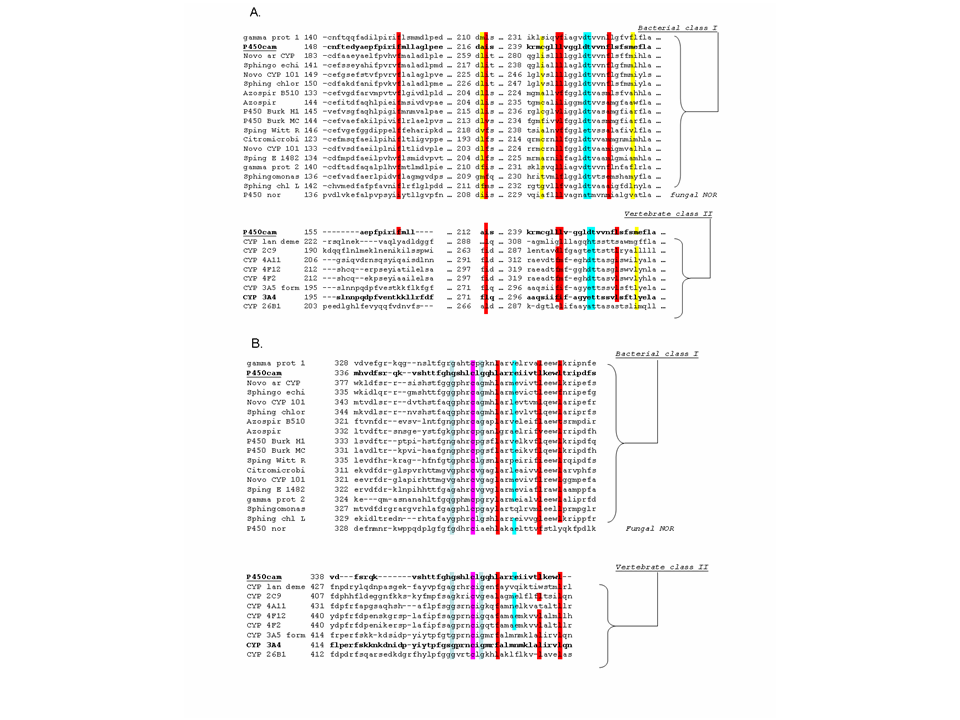

Supplement: Figure S5 — Alignment of microbial cytochromes P450 against P450cam (upper portion) and of vertebrate class II P450s, also against P450cam (lower portion). Microbial sequences used: gamma prot 1 = marine gamma proteobacterium HTCC2207 (ZP_01225512), Novo ar CYP = Novosphingbium aromaticivorans CYP 101D2 (PDB 3NV6), Sphingo echi = Sphingomonas echinoides ATCC14820 (ZP_10341012), Novo CYP 101D1 = a camphor hydroxylase from Novosphingobium aromaticivorans DSM 12444 (PDB 3LXI), Sphing chlor = Sphingomonas chlorophenolicum camphor hydroxylase (ZP_10341012), Azospir B510 = Azospirillium sp. B510 (YP_003451823), Azospir = (BAI74843), P450 Burk H160 = Burkholderia sp. H160 (ZP_03264429), P450 Burk MCO-3 Burkholderia cenocepacia MC0-3 = (YP_001774494), Sping Witt R = Sphingomonas wittichii RW1 (YP_001262244), Citromicrobi = Citromicrobium bathyomarinum JL354 (ZP_06860768), Novo CYP 101 = Novosphingobium aromaticivorans DSM12444 CYP 101C1 (PDB 3OFT_C), Sping E 14820 = Sphingomonas echinoides ATCC 14820 (ZP_10339023), gamma prot 2 = marine gamma proteobacterium NOR51-B (ZP_04956740), Sphingomonas = Sphingomonas sp. KC8 (ZP_09138048), Sphing chl L = Sphingobium chlorophenolicum L-1 (YP_004553185), P450 nor = Cytochrome P450nor from Fusarium oxysporum (BAA03390). Vertebrate P450s: Cyp lan deme = lanosterol 14-α demethylase isoform 1 precursor Homo sapiens (NP_000777), CYP 2C9 = human liver limonene hydroxylase (P11712), CYP 4A11 Homo sapiens (NP_000769), CYP 4F12 = fatty acyl Ω-hydroxylase Homo sapiens (NP_076433), CYP 4F2 = leukotriene-B(4) omega-hydroxylase 1 precursor Homo sapiens (NP_001073), CYP 3A5 form 1 = CYP 3A5 isoform 1 Homo sapiens (NP_000768), CYP 3A4 = CYP 3A4 isoform 1 Homo sapiens (NP_059488), CYP26B1 = retinoic acid hydroxylase Homo sapiens (NP_063938). (TIF) [file pone.0061897.s005.tif]

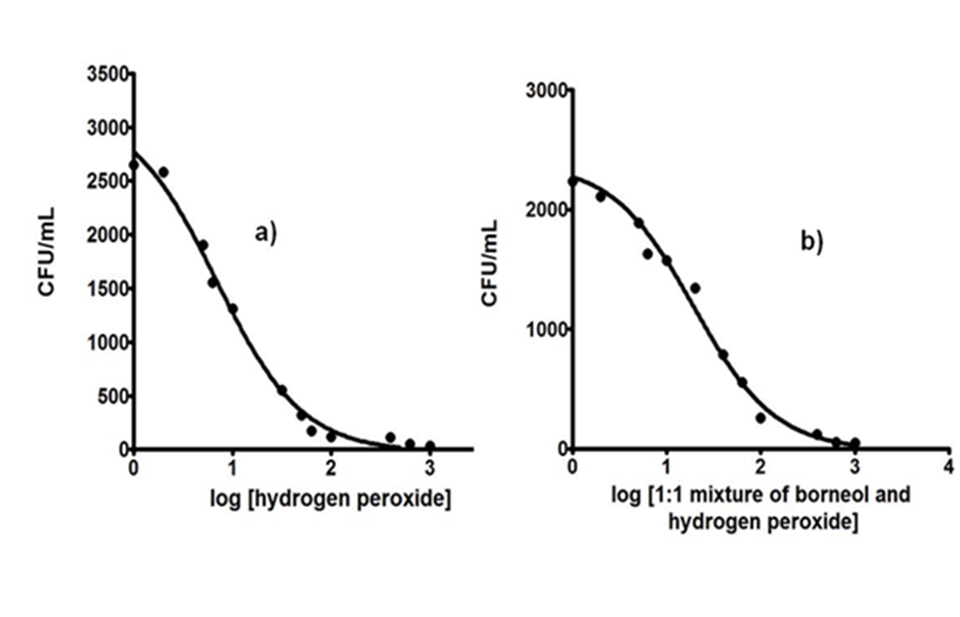

Supplement: Figure S6 — IC50 determination of a) H2O2 and b) of a 1∶1 (molar) mixture of borneol and H2O2 against E. coli , a species of bacterium that lacks cytochrome P450. (TIF) [file pone.0061897.s006.tif]

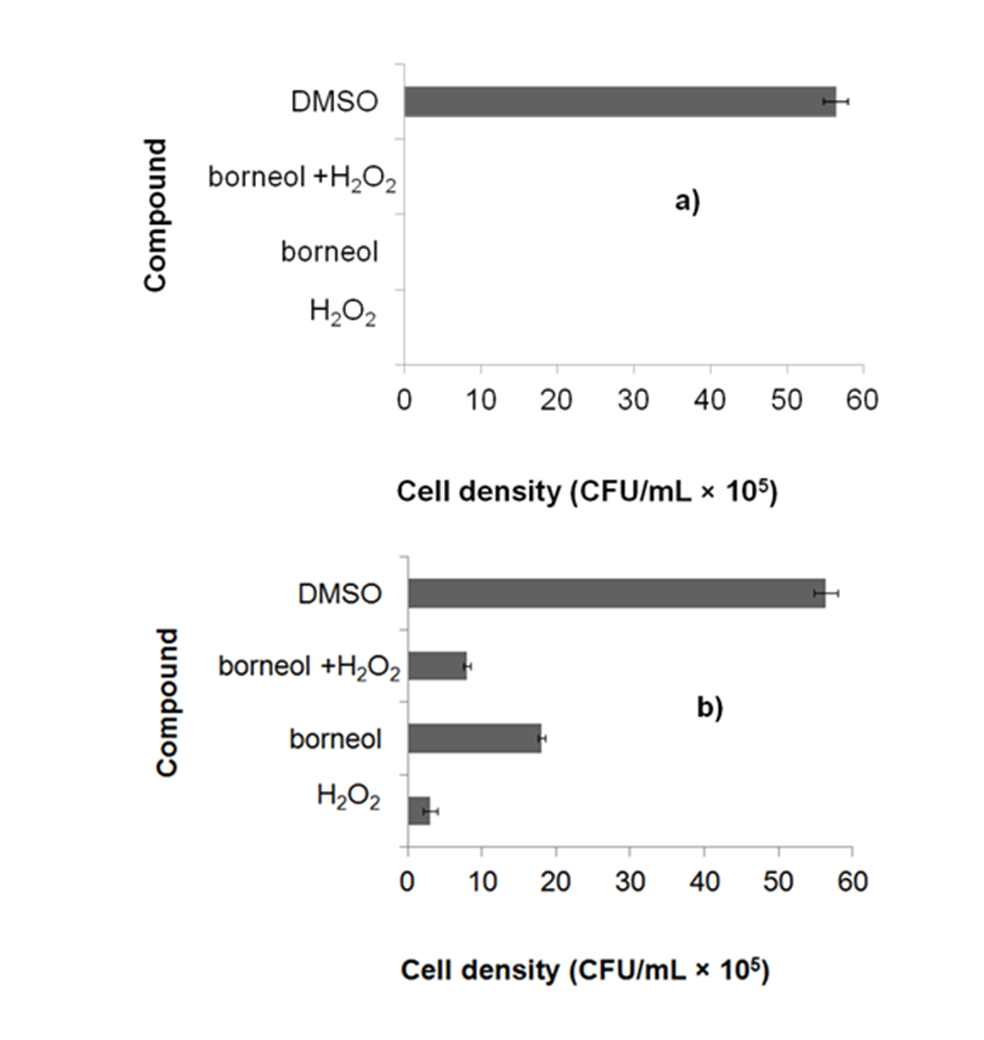

Supplement: Figure S7 — Effect of 16 h incubation of stationary E. coli (a) and P. putida (b) cultures with borneol: H2O2 (1∶1), borneol, or H2O2 (1 mM). (TIF) [file pone.0061897.s007.tif]

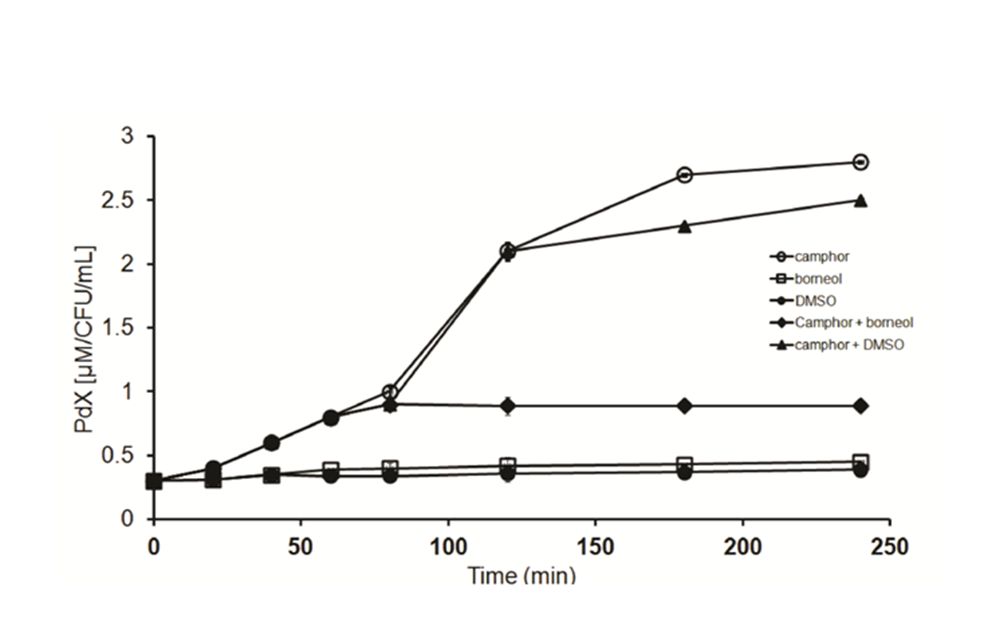

Supplement: Figure S8 — Expression profile of PdX in P. putida , in the presence and absence of camphor or borneol (see experimental map and symbols in Fig. 7a ). Points represent the average ± S. E. of three replicates. (TIF) [file pone.0061897.s008.tif]

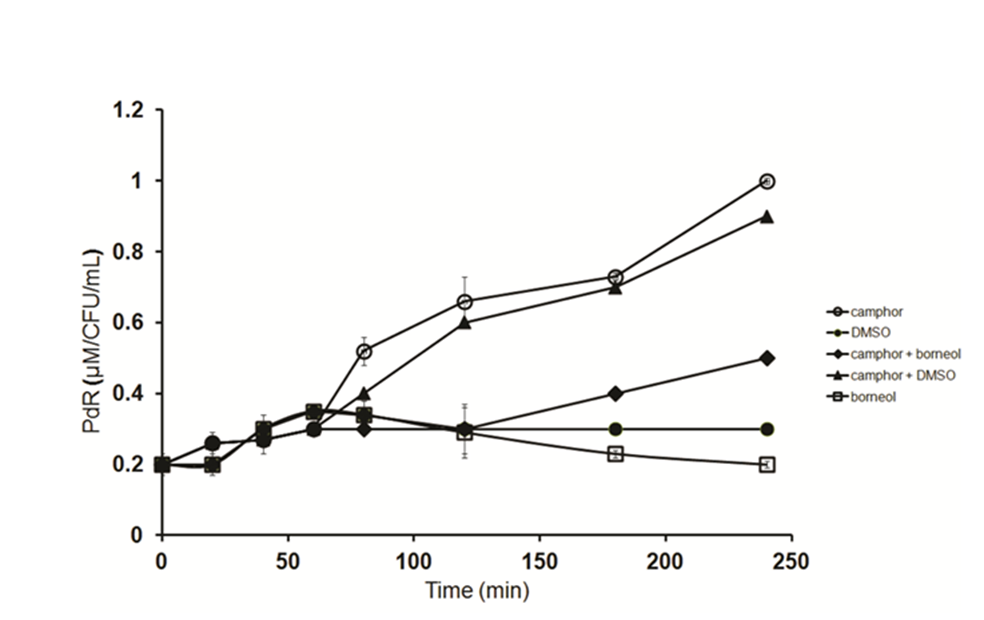

Supplement: Figure S9 — Expression profile of PdR in P. putida , in the presence and absence of camphor or borneol (see experimental map and symbols in Fig. 7a ). Points represent the average ± S. E. of three replicates. (TIF) [file pone.0061897.s009.tif]
