# Supplementary material for: Water Oxidation by a Cytochrome P450: Mechanism and Function of the Reaction
Source: PLoS One. 2013 Apr 25;8(4):e61897. doi: 10.1371/journal.pone.0061897 (PMC3636257; doi:10.1371/journal.pone.0061897)
Supplement: Table S1 — Calculated and literature values of P450cam extinction coefficients at selected wavelengths. (DOC) [file pone.0061897.s010.doc]

**Table S1. Calculated and literature values of P450_cam_ extinction coefficients at selected wavelengths.**

| Wavelength (nm) | Calculated extinction coefficient, (mM^-1^cm^-1^) | Literature extinction coefficient, (mM^-1^cm^-1^) ^[1]^ |
| --- | --- | --- |
| 280 | 64.4 | 63.3 |
| 392 | 68.6 | 102 |
| 410 | 71.1 | 86.5 |
