# Supplementary material for: Water Oxidation by a Cytochrome P450: Mechanism and Function of the Reaction
Source: PLoS One. 2013 Apr 25;8(4):e61897. doi: 10.1371/journal.pone.0061897 (PMC3636257; doi:10.1371/journal.pone.0061897)
Supplement: Table S2 — Formation of 2-D-borneol and 5-ketocamphor in D2O buffer, with the full P450cam system and with the shunted P450cam. (DOC) [file pone.0061897.s011.doc]

**Table S2.** Formation of 2-D-borneol and 5-ketocamphor in D_2_O buffer, with the full P450_cam_ system and with the shunted P450_cam_.

| Assay condition | D-borneol (nmol min^-1^ nmol^-1^ P450) | 5-ketocamphor (nmol min^-1^ nmol^-1^ P450) | NADH consumed (nmol min^-1^ nmol^-1^ P450) | H_2_O_2_ (nmol min^-1^ nmol^-1^ P450) |
| --- | --- | --- | --- | --- |
| rP450_cam_ + rPdX + rPdR + NADH + camphor (pD=7.4)^1, 2^ | 6 ± 3 | 53 ± 24 | 562 ± 230 | ND |
| rP450_cam_ + *m*-CPBA + camphor (pD=7.4)^1, 3^ | 6 ± 3 | ND | N/A | 57.4 ± 10.4 |

Values are average of 4 replicates ± S.E. ND = Not Detected. N/A = Not Applicable.

^1^ Assays were performed with the recombinant proteins (P450_cam_, PdX and PdR)*.* ^2^ NADH and camphor were added to the deuterated phosphate buffer (pD=7.4), treated with O_2_. ^3^ Assays were performed with the recombinant proteins. Shunt agent *m*-CPBA and camphor were added to the deuterated phosphate buffer (pD = 7.4).
