# Supplementary material for: Water Oxidation by a Cytochrome P450: Mechanism and Function of the Reaction
Source: PLoS One. 2013 Apr 25;8(4):e61897. doi: 10.1371/journal.pone.0061897 (PMC3636257; doi:10.1371/journal.pone.0061897)
Supplement: Table S3 — Assays with recombinant proteins at selected temperatures. Formation of borneol, D-borneol, under shunt conditions with the addition of m- CPBA. (DOC) [file pone.0061897.s012.doc]

**Table S3.** Assays with recombinant proteins at selected temperatures. Formation of borneol, D-borneol, under shunt conditions with the addition of *m-*CPBA.

| Temperature (°C) | v_H_ (nmol of borneol/min/nmol P450)^1^ | v_D_ (nmol of D-borneol/min/nmol P450)^1^ | v_H_/v_D_^2^ |
| --- | --- | --- | --- |
| 0 | 148 ± 5 | 2.5 ± 0.5 | 59.2 ± 1.9 |
| 5 | 128 ± 18 | 2.2 ± 0.8 | 58.2 ± 20.8 |
| 10 | 147 ± 39 | 2.5 ± 0.8 | 58.8 ± 20.8 |
| 15 | 166 ± 32 | 2.8 ± 0.3 | 59.3 ± 13.6 |
| 20 | 244 ± 33 | 4.3 ± 0.5 | 56.7 ± 10.1 |

^1^ Data represent the average ± S.E. of 4 replicates. ^2^ Ratios were calculated from the averages. The random errors were calculated by the formula (
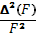

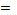

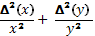
 ) wherein F denotes v_H_/v_D_, x and y denote v_H_ and v_D_. ∆F, ∆x and ∆y denote their corresponding errors.
