# Supplementary material for: Water Oxidation by a Cytochrome P450: Mechanism and Function of the Reaction
Source: PLoS One. 2013 Apr 25;8(4):e61897. doi: 10.1371/journal.pone.0061897 (PMC3636257; doi:10.1371/journal.pone.0061897)
Supplement: Table S4 — Assays with recombinant P450cam, shunted with m -CPBA in H2O and D2O at selected temperatures. Formation of H2O2 or D2O2. (DOC) [file pone.0061897.s013.doc]

**Table S4**. Assays with recombinant P450_cam_, shunted with *m*-CPBA in H_2_O and D_2_O at selected temperatures. Formation of H_2_O_2_ or D_2_O_2_.

| Temperature  (°C) | v_H_ (nmol of H_2_O_2_/min/nmol P450)^1^ | v_D_ (nmol of D_2_O_2_/min/nmol P450)^1^ | v_H_/v_D_ |
| --- | --- | --- | --- |
| 0 | 266 ± 112 | 138 ± 16 | 1.9 ± 0.8 |
| 5 | 647 ± 93 | 106 ± 6 | 6.1± 0.9 |
| 10 | 589 ± 104 | 178 ± 31 | 3.3 ± 0.8 |
| 15 | 363 ± 105 | 178 ± 31 | 2 ± 0.6 |
| 20 | 183 ± 14 | 57 ± 10 | 3.2 ± 0.6 |

^1^ Values are average of 4 replicates ± S.E.
