# Supplementary material for: Water Oxidation by a Cytochrome P450: Mechanism and Function of the Reaction
Source: PLoS One. 2013 Apr 25;8(4):e61897. doi: 10.1371/journal.pone.0061897 (PMC3636257; doi:10.1371/journal.pone.0061897)
Supplement: Table S5 — Formation of borneol and hydrogen peroxide from the P450 catalytic cycle using several shunt agents. (DOC) [file pone.0061897.s014.doc]

**Table S5. Formation of borneol and hydrogen peroxide from the P450 catalytic cycle using several shunt agents.**

| Shunt agent | Borneol (nmol min^-1^ nmol^-1^ P450)^1^ | Hydrogen Peroxide (nmol min^-1^ nmol^-1^ P450)^1^ | P value^2^ |
| --- | --- | --- | --- |
| Cumene hydroperoxide | 428 ± 285 | 518 ± 105 | 0.1 |
| Sodium periodate (NaIO_4_) | 750 ± 183 | 697 ± 285 | 0.9 |
| Calcium hypochlorite (bleach) | 775 ± 283 | 988 ± 122 | 0.3 |
| r-P450_cam_ + *m*-CPBA (table 1, entry 3) | 249 ± 28 | 291 ± 29 | 0.1 |
| Ar + rP450_cam_ + *m*-CPBA (table 1, entry 4) | 404 ± 19 | 444 ± 16 | 1.1 |
| O_2_ + rP450_cam_ + *m*-CPBA (table 1, entry 5) | 173 ± 39 | 204 ± 17 | 0.87 |

^1^ Values are average of 4 replicates ± S.E.

^2^ The P value refers to the Kruskal-Wallis test between borneol and H_2_O_2_ formation rates.
