# Supplementary material for: Water Oxidation by a Cytochrome P450: Mechanism and Function of the Reaction
Source: PLoS One. 2013 Apr 25;8(4):e61897. doi: 10.1371/journal.pone.0061897 (PMC3636257; doi:10.1371/journal.pone.0061897)
Supplement: Material S1 — Supporting information for this paper. This file contains detailed descriptions of various assays, calculations, sequence alignments and P450cam system induction experiments. In addition, there are 9 supplemental Figures and 5 supplemental Tables. (DOC) [file pone.0061897.s015.doc]

# Supporting Online Material for

# Water oxidation by a cytochrome P450: mechanism and function of the reaction

# Brinda Prasad^1^, Derrick J. Mah^1^, Andrew R. Lewis^1^, Erika Plettner^1*^

Department of Chemistry, Simon Fraser University, Burnaby, BC, Canada – V5A 1S6

*To whom correspondence should be addressed. Email: plettner@sfu.ca

**1. Determination of new P450_cam_ extinction coefficient**

The extinction coefficient of ε_280nm_ can be predicted from specific amino acid residues [1], 5 tryptophan residues [2], 9 tyrosine residues [3] and 8 cysteine residues [4]. This value was determined to be 41.91 mM^-1^cm^-1^ and with the addition of hemin at ε_280nm_ (22.5mM^-1^cm^-1^) [5], the predicted value was 64.41 mM^-1^cm^-1^, which is similar to the literature value of P450_cam_ [1]. Using the literature value of P450_cam_ at ε_280nm_, 63.3 mM^-1^cm^-1^ [1], ε_392nm_ value was calculated using Beer-Lambert Law and was found to be 68.6 mM^-1^cm^-1^ and ε_410nm_ value was calculated to be 71.1 mM^-1^cm^-1^.

**2**. **Description of Enzymatic Assays**

**2.1 Assays with recombinant proteins**

Enzymatic assays were performed in 1 mL of 50 mM phosphate buffer (100 mM K^+^, pH = 7.4) bubbled with air/oxygen. The reaction mixture contained 200 nM P450_cam_, 200 nM PdR, 1000 nM PdX and the substrate camphor (1 mM). NADH consumption was initiated by the addition of 1.25 µL of 200 mM stock and monitored for ten minutes at 340 nm. The reaction was stopped by the addition of 1 mL 7.2 µM indanone in CHCl_3_ for the extraction of the product(s) and the substrate. Indanone was the internal standard for the quantitation of products by gas chromatography / mass spectrometry (GC-MS). A second extraction of the aqueous phase with 0.7 mL of CHCl_3_ / indanone followed. The combined organic layers were dried over MgSO_4_ and analysed by GC-MS.

In assays involving *m-*CPBA as a shunt agent for the catalytic cycle, the reaction mixture contained P450_cam_ (180 nM), *m-*CPBA (1 mM), and the substrate camphor (1 mM) in Ar-sparged phosphate buffer (50 mM K^+^, pH = 7.4). The reaction mixture was incubated for 15 minutes at 22 °C and extracted as described previously. The dried organic layers were analysed by GC-MS. (Table 2) In assays with catalase under shunt conditions, an initial stock of 230 units/mL was prepared and 100 µL of this was added to the 1 mL reaction mixture which contained P450_cam_ (180 nM), *m-*CPBA (1 mM), and the substrate camphor (1 mM). The glucose oxidase obtained from Sigma contained catalase (≤ 10 units/mg) and in our *in vitro* assays, an initial stock of 100 units/mL was prepared, and 100 µL of this was added to the 1 mL reaction mixture containing the same components as described before, in addition to glucose (5.5 µM). In experiments with EDTA, an initial stock of 0.684 mM was prepared and 0.1 µL of this was added to the 1 mL reaction mixture which contained P450_cam_ (180 nM), *m-*CPBA (1 mM), and the substrate camphor (1 mM). Butylated hydroxytoluene (BHT) was prepared as a 4.5 mM stock, of which 1 µL was added to the reaction mixture containing the same components as described before.

In experiments that tested for Fenton’s reaction, the reaction mixture (1 mL) contained 180 nM of FeSO_4_, camphor (1 mM) and *m*-CPBA (1 mM) in phsophate buffer (50 mM K^+^, pH = 7.4).

*Steady-state kinetics:* Enzymatic assays were performed under shunt conditions (as mentioned above) to determine the *K*_M_ and *k*_cat_ for formation of borneol and 5-ketocamphor in normal and D_2_O buffers. 1 mL of 50 mM phosphate buffer (100 mM K^+^, pH = 7.4 or pD 7.4) contained camphor at varied concentrations ranging from 30 µM to 2500 µM, and 180 nM P450_cam_. 1 mM shunt agent (*m*-CPBA) was added to the reaction mixtures containing camphor concentrations 500 µM to 2500 µM and to the rest of the concentrations, *m*-CPBA was optimised to a 1:1 ratio with the substrate concentration to avoid the destruction of the heme in P450. The reactions for 5-ketocamphor formation were carried out using vials fitted with PTFE septa and pressurized with pure O_2_. The reaction mixtures were incubated for 20 minutes at room temperature and extracted with CHCl_3_/indanone and analysed by GC-MS.

**2.2 Dependence of the H_2_O_2_ (^17^O) chemical shift on pH**

We observed that the pH of the assay mixtures dropped from 7.4 to 6.3 after 30 minutes of incubation. The pK_a_ of *m*-CPBA is 7.6 [6]. The drop in pH likely comes from the *meta*-chlorobenzoic acid formed during the shunt reaction. Chemical shifts in ^17^O NMR are reported to be dependent on pH and temperature [7]. The chemical shift of the H_2_^17^O_2_ standard prepared by the electrolysis of phosphate buffer (made from H_2_^17^O) was 178 ppm (at a final pH 3.9). The prepared standard was buffered to different pH values ranging from 3-10 and the chemical shift remained constant from pH 3-8 (Supplementary Figs. S1b and c). At pH 10, the chemical shift changed to 194 ppm, due to the formation of HO-O^-^ (Supplementary Fig. S1a). The hydrogen peroxide formed during the borneol cycle had a chemical shift of 178 ppm, matching the chemical shift of fully protonated H_2_^17^O_2_.

**2.3 Reaction in H_2_^17^O buffer, followed by addition of catalase**

Enzymatic assays performed in phosphate buffer (made from H_2_^17^O) (50 mM, 150 mM K^+^, pH 7.4) contained 1 mM camphor, 1 mM *m*-CPBA and recombinant P450_cam_ (0.1 µM) and were incubated for 12 h to detect the formation of H_2_^17^O_2_. To this assay mixture, P450_cam_ (0.02 µM) and *m*-CPBA (0.2 mM) were added at 2 h intervals, to form detectable amounts of H_2_^17^O_2_. A new resonance was observed at 178 ppm, (Fig. 2b(i)) which matched the chemical shift of H_2_^17^O_2_ reported in the literature [8] and of our prepared standard [9]. Controls (in the absence of *m*-CPBA, enzyme or substrate) were run simultaneously, and this resonance was not detected (Figs. 2b(ii), 2b(iii) and 2b(iv)), which led us to conclude that the new peak could not have come from the hydrolysis of *m*-CPBA. Catalase (20 units) was added to the reaction, and the mixture was scanned for the same amount of time as above. The resonance at 178 ppm disappeared (Supplementary Fig. S2 b), confirming that the 178 ppm resonance is due to H_2_^17^O_2_.

**2.4 Assays in D_2_O buffer**

Assays were performed in 1 mL 50 mM phosphate buffer (made from D_2_O) of pD 7.4. The pH of the buffer was adjusted according to the equation: pD = pH meter reading + 0.4. [10]. For assays with the complete P450_cam_ system, the reaction mixture contained: P450_cam_ (180 nM), PdX (900 nM), PdR (180 nM), NADH (0.6 mM), camphor (1.2 mM), and O_2_ was bubbled into the mixture. For assays with shunted P450_cam_, the reaction mixture included camphor (1.25 mM), *m-*CPBA (1 mM), and P450_cam_ (180 nM). Reactions were incubated at 22 °C. The reaction was stopped by the addition of 1 mL 7.2 µM indanone in CHCl_3_ for the extraction of the product(s) and the substrate. A second extraction of the aqueous layer was done with 0.7 mL of CHCl_3_/indanone. The combined organic layers were dried over MgSO_4_ and analysed by GC-MS. Supplementary Table S2 shows the result of assays with the full system and the shunted P450. For ^2^H NMR, 34 µg of 2-D-borneol were collected from pooled extracts of assays that had been run in D_2_O. The sample was dissolved in CHCl_3_ (300 µL) and 200 µL of the solution was transferred to a 3 mm Bruker MATCH nmr tube.

Assays with shunted P450_cam_ were also performed at 0 °C, 5 °C, 10 °C, 15 °C in 1 mL of non-aerated, 50 mM phosphate buffer, in D_2_O or H_2_O, at pD or pH 7.4. Mixtures contained 200 nM P450_cam_, camphor (1.25 mM), and *m-*CPBA (1 mM). Samples were collected after 30, 60, 120 and 360 min and checked by GC-MS for the formation of products. Controls contained the reaction mixtures in the absence of the substrate, enzyme or shunt agent. Figure 3a shows the ratios of the product formation velocities in H_2_O/D_2_O (v_H_/v_D_) (Supplementary Tables S3 and S4).

**2.5 Assays with human P450 (CYP3A4)**

To test whether a different camphor-metabolizing P450 is also able to reduce camphor to borneol under low oxygenation, we reacted a human cytochrome P450, (CYP3A4) containing NADPH and the cognate reductase with camphor under shunt conditions. For shunt, the enzymatic assays were performed in 1 mL 50 mM phosphate buffer (150 mM K^+^) of pH 7.4. The reaction mixture included the substrate camphor (1.25 mM), *m-*CPBA (1 mM), and human P450 CYP3A4 (0.5 pmol). The reaction mixture was incubated for 20 min at 22 °C. Controls contained the reaction mixtures in the absence of the substrate, enzyme or shunt agent. The reaction was stopped by the addition of 1 mL 7.2 µM indanone in CHCl_3_ for the extraction of the product(s) and the substrate. A second extraction of the aqueous layer was done with 0.7 mL of CHCl_3_/indanone. The combined organic layers were dried over MgSO_4_ and analysed by GC-MS.

In the enzymatic assays under highly oxygenated conditions, no detectable products were obtained. One possible explanation is that CYP3A4 exhibits cooperativity with certain substrates [11] and is allosterically activated with others [12]. Shunting with *m*-CPBA, the enzyme produced 18.6 ± 0.6 µmol/min/µmol P450 of 5-*exo*-hydroxy camphor, but no detectable borneol. Therefore, the borneol formation reaction is not general to camphor-metabolizing cytochromes P450.

**2.7 Assays with other shunting agents**

Enzymatic assays were performed in 50 mM phosphate buffer (150 mM K^+^, pH 7.4), with recombinant P450_cam_ (180 nM), substrate camphor (1.25 mM) and one of the shunt agents (cumene hydroperoxide, sodium periodate, *m*-CPBA or calcium hypochlorite (bleach)) added at a final concentration of 1 mM.

The reaction was stopped by the addition of 1 mL 7.2 µM indanone in CHCl_3_ for the extraction of the product(s) and the substrate. A second extraction of the aqueous layer was done with 0.7 mL of CHCl_3_/indanone. The combined organic layers were dried over MgSO_4_ and analysed by GC-MS (Supplementary Table S5).

**2.8 Assay with dithionite**

To test whether the reduced form of P450_cam_ reduces camphor, P450_cam_ (180 nM) was incubated in 1 ml phosphate buffer (50 mM, 150 mM K^+^) with camphor (1 mM) and dithionite (1 mM). The reaction was stopped, extracted and analyzed as described above in section 2.4. Reduced P450_cam_, alone with camphor or with its redox partners and camphor, did not generate borneol in detectable amounts (Supplementary Fig. S3a).

**2.9 Thermodynamic calculations for hydroxylation and the borneol cycle**

The mechanism presented in Fig. 4 was divided into six steps, and the contribution from each step was calculated using literature data, if available (Supplementary Fig. S4a). For the estimation of the heat of formation of borneol (see step IV below), calculations were done on the MOPAC interface of Chem 3D (v. 11), using the AM-1 method, the closed shell (restricted) wave function, the optimizer EF and water as solvent. All atoms were allowed to move freely, to a minimum RMS of 0.1. Heats of formation were estimated, using that program, for both camphor in water and borneol in water.

Step I: H_2_O(*_l_*) 🡪 H**^•^**_(aq)_ + OH**^•^**_(aq)_

The reduction potential of OH^•^ + H^+^ + e^-^ 🡪 H_2_O(*_l_*) has been estimated by Koppenol and Liebman as ε_o_ = 2.59 ± 0.04 V. This yields a ∆G_o_ value of -250 ± 4 kJ/mol. Step I is the reverse, or 250 ± 4 kJ/mol [13].

Step II: Cpd I + H**^•^** 🡪 Cpd II-H (= protonated Cpd II, **13**, Fig. 4)

The properties of Cpd II-H have been estimated by Green, Dawson and Gray. Based on the one-electron reduction potential of Cpd I (ε_o_I = 1.3 ± 0.1) and the pK_a_ of Cpd II-H, they estimated the ∆G_o_ dissociation of Cpd II 🡪 Cpd I + H**^•^** to be 98 ± 4 kcal/mol (= 410 ± 17 kJ/mol). Step II is the reverse, or -410 ± 17 kJ/mol [14].

Step III: OH**^•^** + H_2_O 🡪 H_2_O_2_ + H**^•^**

We have estimated this as follows: From Koppenol and Liebman we know that Step I is 250 kJ/mol. The heat of formation of H_2_O(*_l_*) is -285.8 kJ/mole [15]. The heat of formation of H^•^ is listed as +218 kJ/mol [15]. Therefore, based on the energy of Step I, and assuming that most of this energy comes from enthalpy contributions [16], we estimate the heat of formation of OH**^•^**_(aq)_ as -253.8 kJ/mol.

This value now enables us to estimate the ∆H_o_ for Step III, using the listed heat of formation for H_2_O_2_ of -187.8 kJ/mol [15] and this works out to be +569.8 kJ/mol.

Step IV: Camphor + H**^•^** 🡪 borneol radical

For this step, we first had to estimate the heat of formation of the borneol radical. The heat of formation of camphor has been measured as -76.3 ± 0.6 kcal/mol = -319 ± 2 kJ/mol [17]. The heat of formation of borneol has not been measured, to our knowledge and, therefore, we estimated it from the heat of formation of camphor combined with semi-empirical calculations. Both, camphor and borneol were energy minimized, and their heats of formation were estimated, as described in the general section above. For camphor, we obtained “H_f_” = -47.89 kcal/mol (-200.3 kJ/mol) and for borneol we obtained “H_f_” = -66.68 kcal/mol (-278.9 kJ/mol). These results make sense, since camphor is a strained ketone, and this strain is somewhat relieved in borneol. Also, both compounds were energy-minimized in water, and borneol should be better solvated than camphor. The difference between these two calculated values is: “∆H_f_” = -78.6 kJ/mol. In our experience with that software, these estimates have errors of approximately 4 kJ/mol. Therefore, borneol should be 78.6 kJ/mol more stable than camphor, which gives us a ∆H_o_(borneol) = ∆H_o_(camphor) – 78.6 kJ/mol ≅ -398 ± 6 kJ/mol.

Now the ∆H_f_ of the borneol radical can be estimated from the following equation:

Borneol 🡪 borneol radical + H**^•^**

which is a C-H bond dissociation reaction. Detailed listings of C-H bond dissociation energies have been compiled by Blanksby and Ellison [16]. They list primary C-H bond dissociation energies as 423 ± 2 kJ/mol and secondary C-H bond dissociation as 392 kJ/mol. We have chosen the higher value to get an upper estimate because the C-H that is α to the OH group in borneol should be more polarized than a hydrocarbon C-H and, therefore, slightly more difficult to break homolytically.

From the values for borneol (-398 ± 6 kJ/mol) and H**^•^** (218 kJ/mol) we estimate that the borneol radical has ∆H_o_ ≅ -180 ± 8 kJ/mol.

Now the ∆H_o_ of Step IV can be estimated from camphor (-319 ± 2 kJ/mol), H**^•^** (218 kJ/mol) and borneol radical (-180 ± 8 kJ/mol), and this gives ∆H_o_ = -79 ± 10 kJ/mol.

Step V: is the reverse of Step II and, therefore has a ∆H_o_ ≅ 410 ± 17 kJ/mol, provided entropy contributions are negligible.

Step VI: This step is a C-H bond formation reaction; the C-H bond dissociation in reverse. To be consistent with our previous choice (Step III), we used -423 ± 2 kJ/mol.

Net reaction.

Overall, using all the steps of Born-Haber cycle, we obtain a net ∆H_o_ = 318 ± 16 kJ/mol for the borneol cycle.

The value of ∆H_o_ = H_products_ – H_reactants_ = (-187.8 + -398) – (-571.6 + -319) = 305 ± 8 kJ/mol.

The borneol cycle could end in two ways (Supplementary Fig S4 b): i) the oxidation of an enzymatic residue by Cpd I or ii) the abstraction of a H-atom from an additional water molecule by the borneol radical and the “rebinding” of the resulting OH^•^ to the OH^•^ bound on Cpd II-H. This would result in a second molecule of H_2_O_2_. Both proposed termination reactions lead to the ferrous P450.

**2.10 Alignment of cytochromes P450 and superposition of CYP101A1 (P450_cam_) and CYP3A4**

A search, using the Basic Local Alignment Search Tool (BLAST) on the National Center for Biotechnology (NCBI) web site (<http://www.ncbi.nlm.nih.gov/sutils/blink.cgi?mode=query>) was performed, using the P450_cam_ protein sequence as the reference. The search was done among all non-redundant GenBank CDS translations+PDB+SwissProt+PIR+PRF excluding environmental samples from WGS projects, using BLASTP 2.2.26+.

The BLAST search of the P450_cam_ protein sequence revealed related CYPs (19, with ≥ 40% sequence identity, and 50 with ≥ 30 but < 40% sequence identity), all from soil and marine bacteria. Protein sequences (16 in total) were then chosen, along with P450nor, a distant fungal CYP from *Fusarium oxysporum* (locus1XQD_A), that was not detected in the BLAST search and that catalyzes the reduction of nitric oxide. [18] These protein sequences were then subjected to exhaustive pairwise alignment, using Neighbor-joining phylogeny in SECentral (Align Plus 4.0, Scientific and Educational Software, Durham, NC, USA). The result of this alignment is shown in the upper portion of Fig S5.

It is important to note that the DT pair (251 and 252) and E366 in P450_cam_ are highly conserved among these CYPs. These are the residues known to hold the water molecules above the porphyrin. In addition, the O_2_ tunnel is conserved, with hydrophobic residues. For example, F (163 in P450_cam_) is highly conserved, A167 in P450_cam_ is either A, V, I, M or L in the others, I220 in P450_cam_ is I, V, F or M in the others, A219 in P450_cam_ is A, L, V, M or F in the others and L245 in P450_cam_ is L, V or I in the others. Hydrophobic residues from the crevasse in P450_cam_, that are also hydrophobic in all the others, include the residues that align with L257, M261, P278, I281, L371 and L375 of P450_cam_. Thus, it is possible that the water oxidation, coupled to the reduction of an organic substrate, is widespread among bacteria in the environment.

A similar exhaustive pairwise alignment was done with eight vertebrate class II P450s and P450_cam_ as the reference. The results are in the lower portion of Fig. S5. Of note is the poor alignment around F163 of P450_cam_: the vertebrate proteins have either K or A there; CYP3A4 has K. Residue F163 is part of the hydrophobic O_2_ binding site of P450_cam_ that is near the porphyrin. This difference prompted us to superimpose the three dimensional structures of P450_cam_ and CYP3A4.

Structures were visualized, superimposed and docked in Molecular Operating Environment (MOE). Briefly, the PDB structures were opened in MOE and prepared by calculating the protonation of each residue at pH 7.0, 300K and 0.1 M salt, and assigning charges according to the default settings under “Compute|Protonate 3D”. For superposition, the two proteins were first aligned, using the “Align” protocol, then they were superimposed, with P450_cam_ as the reference, with “all residues” and “accent secondary structure matches” selected. The results from superposition are discussed in the main text.

**2.11 Toxicity assays of borneol, hydrogen peroxide with *E. coli* and *P. putida***

The IC_50_ of hydrogen peroxide (Supplementary Fig. S6a) and of a 1:1 mixture of borneol and hydrogen peroxide (Supplementary Fig. S6c)were determined for *E. coli* as reported previously [19] and found to be 7 nM and 17 nM, respectively whereas H_2_O_2_ was found to be less toxic to *P. putida* (IC_50_ was found to be 48 nM (Supplementary Fig. S6b). In comparison, the IC_50_ of borneol alone was 19.3 nM for *E. coli* [19].

The effect of borneol:H_2_O_2_ (1:1 molar ratio of 1 mM concentration each), borneol, or H_2_O_2_ on the survival of *P. putida* (strain ATCC 17453) and *E. coli* (strain XL-1 BL) with the stationary cultures of these two strains was performed in the same way as described previously [19].

The mean cell densities of *E. coli* and *P. putida* stationary cultures that had been incubated with borneol, H_2_O_2_ or a 1:1 mixture of borneol and H_2_O_2_ for 16 h, revealed a significant effect of treatment (*ANOVA* for *P. putida*, *P* < 0.0001; *F* = 8.93 and for *E. coli*, *P* < 0.0001; *F* = 12.07). Turkey-Kramer pairwise comparisons of borneol, hydrogen peroxide, 1:1 mixture of borneol + hydrogen peroxide, and the control (dimethyl sulphoxide, DMSO) for each bacterial species revealed that hydrogen peroxide or the 1:1 mixture of borneol + hydrogen peroxide are bacteriostatic to *P. putida* (DMSO/hydrogen peroxide: *P* < 0.01; DMSO/borneol: *P* < 0.01; DMSO/borneol + hydrogen peroxide: *P* < 0.01). In contrast, borneol, hydrogen peroxide and the 1:1 mixture were highly toxic (DMSO/hydrogen peroxide: *P* < 0.01; DMSO/borneol: *P* < 0.01; DMSO/borneol + hydrogen peroxide: *P* < 0.01) to *E. coli* (no colonies were observed, Supplementary Fig. S7). This difference in the toxicity of the borneol cycle products on the producer, compared to a bacterial species that lacks P450, may be an adaptive advantage for *P. putida* during times of low aeration, when camphor resources cannot be metabolized but borneol has been produced.

**2.12 The effect of camphor, borneol and DMSO on the expression of P450_cam_, PdX, and PdR**

To study the effect of borneol on the induction of the P450_cam_ system, we grew *P. putida* for 7 generations at 27 °C without camphor under aerated conditions (O_2_ concentration 5 mg/mL), and then divided the culture into three equal portions. Camphor (0.03g in 300 µL DMSO), borneol (0.03g in 300 µL DMSO) or the control (DMSO), was added to one of the three portions, and the cultures were incubated at 27 °C. Samples collected at regular intervals up to 4 h were harvested at 10,000 × g for 90 seconds at 4 °C. The entire experiment was performed in 3 replicates. Cultures induced with camphor were divided into three portions after 60 minutes and borneol (0.015g in 300 µL DMSO), DMSO, or no compound/solvent was added to one of the three portions (Fig. 7a). Lysis of the cultures was performed as described previously [20]. The clarified lysate samples obtained at regular time intervals were scanned from 200-700 nm and the concentrations of P450_cam_, PdR, and PdX were obtained from the characteristic absorbances. The extinction coefficients we have determined for purified P450_cam_ (Supplementary Table S1) were used to estimate the concentration of P450_cam_ in the lysates. In order to correct for the increase in absorbance due to bacterial growth, the enzyme concentration was divided by the number of colony forming units obtained for that time point.

Using Graphpad prism software, the Kruskal-Wallis test was chosen to compare the effects of 5 groups (camphor, borneol, DMSO, camphor + borneol, camphor + DMSO). The effect of camphor was significant on the expression of P450_cam_ after 80 minutes (*P* < 0.01). The DMSO treatments differed significantly at each time point after induction from the camphor-induced treatments (*P* < 0.01).

*ANOVA* (Tukey-Kramer test) was performed within each time series of the three replicates for each treatment. Camphor showed a significant effect of increasing the P450 expression (*P* < 0.01), 80 min after its addition to the culture (marked * in Fig. 7). The addition of DMSO to the camphor-induced cultures did not have any effect (P > 0.01 within the DMSO series). The addition of borneol to the camphor-induced culture showed a significant (*P* < 0.01) decrease in expression 80 minutes after its addition to the camphor-induced culture (marked # in Fig. 7). In the borneol- and camphor-treated cultures, the normalized P450_cam_ levels 120 min. after borneol addition were close to the background expression at 0 minutes (Fig. 7b). The results for PdX and PdR are shown below in Supplementary Figures S8 and S9.

**Supporting Information References**

1. Pace CN, Vajdos F, Fee L, Grimsley G, Gray T (1995) How to measure and predict the molar absorption coefficient of a protein. Protein Sci 4: 2411-2423.

2. Prasad S, Mazumdar S, Mitra S (2000) Binding of camphor to *Pseudomonas putida* cytochrome P450_cam_: steady-state and picosecond time-resolved fluorescence studies. FEBS Lett 477: 157-160.

3. Janig GR, Makower A, Rabe H, Friedrich J, Ruckpaul K (1984) Chemical modification of tyrosine residues at the active centre of cytochrome P450_cam_. Biomed Biochim Acta 43: K17-24.

4. Nickerson DP, Wong LL (1997) The dimerization of *Pseudomonas putida* cytochrome P450_cam_: practical consequences and engineering of a monomeric enzyme. Protein Eng 10: 1357-1361.

5. Thompson AM, Reddi AR, Shi X, Goldbeck RA, Moenne-Loccoz P, et al. (2007) Measurement of the heme affinity for yeast dap1p, and its importance in cellular function. Biochemistry 46: 14629-14637.

6. Wolak M, van Eldik R (2007) Mechanistic studies on peroxide activation by a water-soluble iron(III)-porphyrin: implications for O-O bond activation in aqueous and nonaqueous solvents. Chemistry 13: 4873-4883.

7. Boykin D.W. (Ed.), 1981. ^17^O NMR spectroscopy in Organic Chemistry. CRC Press, Boca Raton.

8. Casny M, Rehder D, Schmidt H, Vilter H, Conte V (2000) A ^17^O NMR study of peroxide binding to the active centre of bromoperoxidase from *Ascophyllum nodosum*. J Inorg Biochem 80: 157-160.

9. Prasad B, Lewis AR, Plettner E (2011) Enrichment of H_2_^17^O from tap water, characterization of the enriched water, and properties of several ^17^O-labeled compounds. Anal Chem 83: 231-239.

10. Chaen S, Yamamoto N, Shirakawa I, Sugi H (2001) Effect of deuterium oxide on actomyosin motility in vitro. Biochim Biophys Acta 1506: 218-223.

11. Denisov IG, Baas BJ, Grinkova YV, Sligar SG (2007) Cooperativity in cytochrome P450 3A4: linkages in substrate binding, spin state, uncoupling, and product formation. J Biol Chem 282: 7066-7076.

12. Woods CM, Fernandez C, Kunze KL, Atkins WM Allosteric activation of cytochrome P450 3A4 by alpha-naphthoflavone: branch point regulation revealed by isotope dilution analysis. Biochemistry 50: 10041-10051.

13. Koppenol WH, Liebman JF (1984) The Oxidizing Nature of the Hydroxyl Radical - a Comparison with the Ferryl Ion (FeO^2+^). J Phys Chem 88: 99-101.

14. Green MT, Dawson JH, Gray HB (2004) Oxoiron(IV) in chloroperoxidase compound II is basic: implications for P450 chemistry. Science 304: 1653-1656.

15. Castellan GW (1983) "Physical chemistry" Addison Wesley Publishing Company. 3^rd^ Ed.: p381.

16. Blanksby SJ, Ellison GB (2003) Bond dissociation energies of organic molecules. Acc Chem Res 36: 255-263.

17. Steele WV (1977) J Chem Thermodyn 9: 311-314.

18. Oshima R, Fushinobu S, Su F, Zhang L, Takaya N, et al. (2004) Structural evidence for direct hydride transfer from NADH to cytochrome P450nor. J Mol Biol 342: 207-217.

19. Prasad B, Rojubally A, Plettner E Identification of Camphor Oxidation and Reduction Products in Pseudomonas putida: New Activity of the Cytochrome P450_cam_ System. J Chem Ecol 37: 657-667.

20. Rojubally A, Hua Cheng, S. , Foreman, C. , Huang, J. , Agnes, G. , Plettner, E. (2007) Linking of cytochrome P450_cam_ and putidaredoxin by a co-ordination bridge. Biocatalysis and Biotransformation 25: 301-317.
